# Supplementary material for: Programmable low-cost DNA-based platform for viral RNA detection
Source: Sci Adv. 2020 Sep 25;6(39):eabc6246. doi: 10.1126/sciadv.abc6246 (PMC7518872; doi:10.1126/sciadv.abc6246)
Supplement: abc6246_FileS1.zip [file abc6246_FileS1.zip › File S1/Instruction for the Viral RNA detection target tool.docx]

**Instruction for Choosing Targets of Viral RNA for DNA Nanoswitch Detection**

Harvorsen Lab, Lifeng Zhou et al.

Matlab main function: Viral_RNA_Detection_Project.m, users can develop their own tools based on this file.

Matlab GUI file for this project: ViralRNADetectionTool.m and ViralRNADetectionTool.fig

Instruction for the GUI: Unzip all files into a new folder after downloading the ViralRNADetectionTool.zip from website xxx or xxx. Launch your matlab and navigate to working folder the folder with all codes. Open ViralRNADetectionTool.m and run it. Then use the following procedures to obtain the targeted sequences. For practice, users can use the Example_Viral_A_seq.txt, Example_Backbone_seq.txt, Example_Viral_B_seq.txt and Example_Viral_A2_seq.txt.


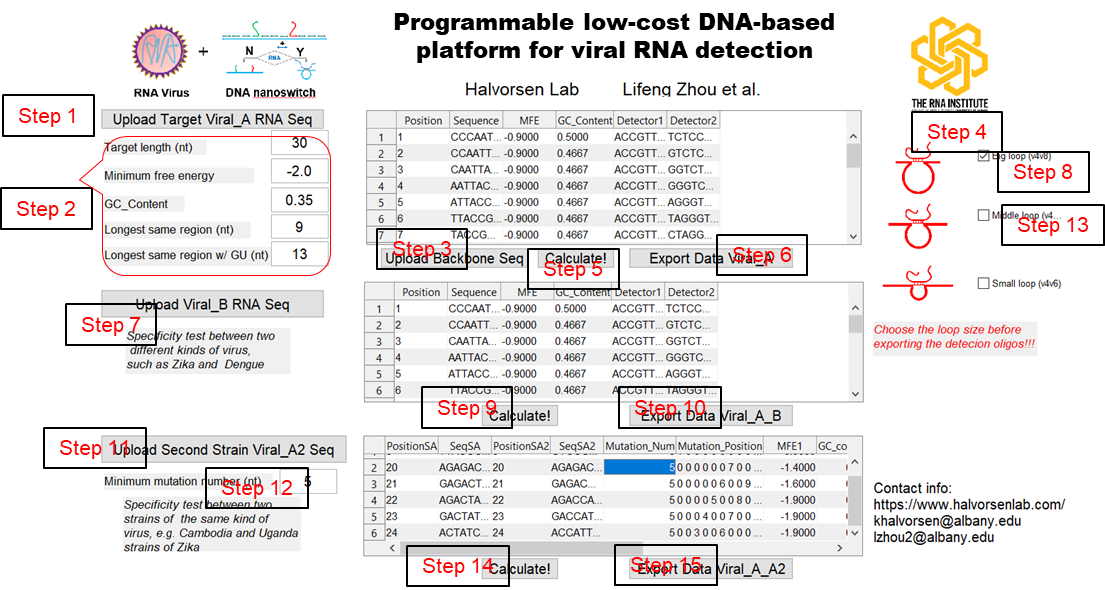


**Fig. I. Graphical user interfaces (GUI) of the tool.**

**Fig. I** shows the graphical user interfaces (GUI) of the tool for choosing the detection sequences of targeted viral RNA for the nanoswitch detection. It was developed in Matlab and the detailed code was named Viral_RNA_Detection_Project.m which can be used to do further development. Here, the functions discussed in the paper were summarized as follows.

**Step 1.** Click ‘Upload Target Viral_A RNA seq’ and a window will pop out where user can find the target viral RNA sequence file. It must be a .txt file and ensure there is no whitespace among the sequence. An example file named *Example_Viral_A_seq .txt* was provided.

**Step 2.** Input the ‘Target length’, ‘Minimum free energy (kcal/mol)’, ‘GC_Content (%)’, ‘Longest same region (nt)’, and ‘Longest same region w/GU (nt)’ in the blank boxes next to them.

*Target length is the length of the targeted region of the viral RNA. It may influence the detection efficiency (supporting information,* ***Fig. S3****) and determines the detect arm length of the nanoswitch. 30 is the recommended for the viral RNA detection.*

*Minimum free energy (kcal/mol) provides hint for the most stable structure that the targeted RNA sequence could form. -2.0 was recommended for the viral RNA detection.*

*GC_Content (%) is the number percentage of G (Guanine) and C (Cytosine) in the entire sequence of the target. In theory, GC base-pair is stronger than the AU base-pair and the more GC base-pair, the more stable the formed duplex. Here, 0.35 was recommended for the viral RNA detection, which means there are at least 11 GC base-pairs in a 30 nt target sequence.*

*Longest same region (nt) (Supporting information Note S1) is used to eliminate the targets that can form stable structures with other regions of the viral RNA or can result in preoccupation of the detect arms by the backbone oligos of nanoswitch. In addition, it is used to eliminate the similar targets on other kinds of viral RNA to achieve high detection specificity.*

*Longest same region w/ GU (nt) (Supporting information Note S1) has similar function as Longest same region (nt). The only difference between them is that the former also takes the wobble guanine-uracil (GU) base-pair into consideration.*

**Step 3.** Click the ‘Upload Backbone Seq’ under the first table. A window will pop out for user to choose the backbone sequence of the Nanoswitch. In the paper, it is M13. For the practice, user can choose the *Example_Backbone_Seq.txt*.

**Step 4.** Choose the loop size of the nanoswitch on the right panel. The loop size of nanoswitch was determined by the positions of the two detection arms (Supporting information, **Fig. S11**).

**Step 5.** Click the ‘Calculate!’ under the first table.

*After the calculation, the result will be shown in the top table where:*

*Position: the position of the target on the viral RNA;*

*Sequence: the sequence of the target candidate, where U is replaced by T;*

*MFE: minimum free energy (kcal/mol);*

*GC_Content: G and C percentage (%);*

*Detector 1: the sequence of the first detector oligo v4 (Supporting information,* ***Fig. S11****);*

*Detector 2: the sequence of the second detector oligo v8 (Supporting information,* ***Fig. S11****).*

**Step 6.** Click ‘Export Data Viral_A’, a window will pop out for users to store the data shown in the top table. The format of the saved data file is .csv.

*For the specificity test of another kind of virus, go to step 6!*

*For the specificity test of another strain of the same kind of virus, go to step 10!*

**Step 7.** Click ‘Upload Viral_B Seq’, a window will pop out for users to choose the other viral RNA sequence. An example file named *Example_Viral_B_seq.txt* was provided. In the paper, it is a Dengue viral RNA.

**Step 8.** Choose the loop size of the nanoswitch on the right panel. (Same as Step 3)

**Step 9.** Click the ‘Calculate!’ under the first table. There will be no window pop out this time.

*After the calculation, the result will be shown in the middle table whose data structure is the same as the top table.*

**Step 10.** Click ‘Export Data Viral_A_B’, a window will pop out for users to store the data shown in the middle table. The format of the saved data file is .csv.

-> For the specificity test of another strain of the same kind of virus,

**Step 11.** Click ‘Upload Second Strain Viral_A2 Seq’, a window will pop out for users to choose the other viral RNA sequence. An example file named *Example_Viral_A2_seq.txt* was provided. In the paper, it is a Uganda strain.

**Step 12.** Input the ‘Minimum mutation number (nt)’ in the blank box.

Minimum mutation number is the number of mutations of the target region between two strains of the same kind of virus (Supporting information, **Fig. S12**).

**Step 13.** Choose the loop size of the nanoswitch on the right panel. (Same as Step 3)

**Step 14.** Click the ‘Calculate!’ under the first table. There will be no window pop out this time.

*After the calculation, the result will be shown in the bottom table whose data structure is as follows.*

*Because the target sequences of the two strains are very similar to each other, both of their information are presented in the table.*


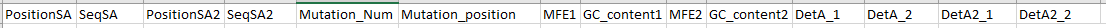


*PositionSA: position of the target on the first strain A;*

*SeqSA: sequence of the target on the first strain A;*

*PositionSA2: position of the target on the second strain A2;*

*SeqSA2: sequence of the target on the second strain A2;*

*Mutation_Num: the mutation number of the target between the two strains;*

*Mutation_position: Show the position of the mutation, e.g., a target with 30 nt,*


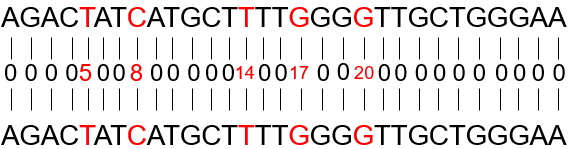


*the Mutation_position is:‘*0 0 0 0 5 0 0 8 0 0 0 0 0 14 0 0 17 0 0 20 0 0 0 0 0 0 0 0 0 0 0 0’ *where ‘0’ means the nucleotides are the same, other numbers mean there are mutations at those positions;*

*MFE1: minimum free energy (kcal/mol) of the SeqSA;*

*GC_content1: G and C percentage (%)of the SeqSA;*

*MFE2: minimum free energy (kcal/mol) of the SeqSA2;*

*GC_content2: G and C percentage (%)of the SeqSA2;*

*DetA_1: the sequence of the first detector oligo v4 of the SeqSA (Supporting information,* ***Fig. S11****);*

*DetA_2: the sequence of the second detector oligo v8 of the SeqSA (Supporting information,* ***Fig. S11****).*

*DetA2_1: the sequence of the first detector oligo v4 of the SeqSA2 (Supporting information,* ***Fig. S11****);*

*DetA2_2: the sequence of the second detector oligo v8 of the SeqSA2 (Supporting information,* ***Fig. S11****).*

**Step 15.** Click ‘Export Data Viral_A_A2’, a window will pop out for users to store the data shown in the middle table. The format of the saved data file is .csv.
