# Supplementary figures and images for: Programmable low-cost DNA-based platform for viral RNA detection
Source: Sci Adv. 2020 Sep 25;6(39):eabc6246. doi: 10.1126/sciadv.abc6246 (PMC7518872; doi:10.1126/sciadv.abc6246)

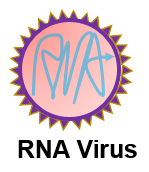

Supplement: abc6246_FileS1.zip [file abc6246_FileS1.zip › File S1/Matlab_Viral_RNA_Detection/RNA virus.PNG]

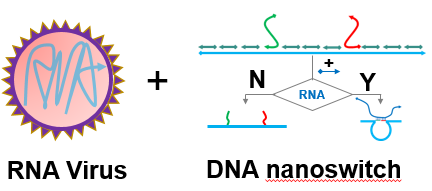

Supplement: abc6246_FileS1.zip [file abc6246_FileS1.zip › File S1/Matlab_Viral_RNA_Detection/RNA virus with NS.PNG]

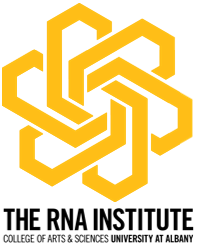

Supplement: abc6246_FileS1.zip [file abc6246_FileS1.zip › File S1/Matlab_Viral_RNA_Detection/RNA institute logo_small.PNG]

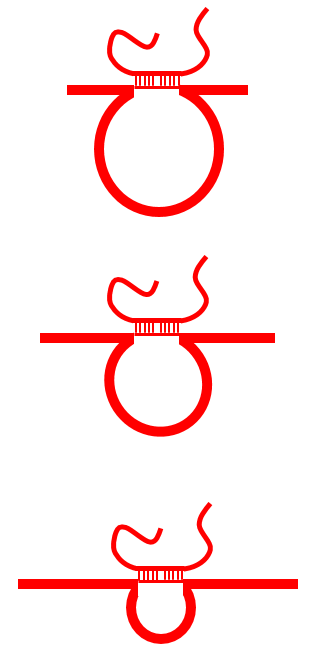

Supplement: abc6246_FileS1.zip [file abc6246_FileS1.zip › File S1/Matlab_Viral_RNA_Detection/NS_three loop.PNG]
