# Supplementary material for: Programmable low-cost DNA-based platform for viral RNA detection
Source: Sci Adv. 2020 Sep 25;6(39):eabc6246. doi: 10.1126/sciadv.abc6246 (PMC7518872; doi:10.1126/sciadv.abc6246)
Supplement: abc6246_SM.pdf [file abc6246_SM.pdf]

Science Advances
AAAS

advances.sciencemag.org/cgi/content/full/sciadv.abc6246/DC1

Supplementary Materials for

Programmable low-cost DNA-based platform for viral RNA detection

Lifeng Zhou, Arun Richard Chandrasekaran, Jibin Abraham Punnoose, Gaston Bonenfant, Stephon Charles, Oksana Levchenko, Pheonah Badu, Cassandra Cavaliere, Cara T. Pager* and Ken Halvorsen*

*Corresponding author. Email: ctpager@albany.edu or khalvorsen@albany.edu

Published 21 August 2020, *Sci. Adv.* **6**, eabc6246 (2020)
DOI: 10.1126/sciadv.abc6246

This PDF file includes:

Figs. S1 to S19
Tables S1 to S10

Other Supplementary Material for this manuscript includes the following:

(available at advances.sciencemag.org/cgi/content/full/sciadv.abc6246/DC1)

File S1

Contents

Figures

Fig. S1. DNA nanoswitch construction and in vitro transcription (IVT) of viral RNA.

Fig. S2. Fragmentation analysis of ZIKV RNA.

Fig. S3. Optimization of detection arm length.

Note S1. Choosing the detection targets of viral RNA.

Fig. S4. Considerations for choosing target sequences of viral RNA.

Fig. S5. Schematic showing assembly of DNA nanoswitch and interference by excess backbone oligos.

Fig. S6. Graphical user interface (GUI) for obtaining potential viral RNA targets.

Fig. S7. Analysis of the 18 DNA nanoswitches designed for ZIKV RNA detection.

Fig. S8. An example of gel image of the 18 mixed nanoswitches detection sensitivity test.

Fig. S9. Detection sensitivity test of single nanoswitch.

Fig. S10. Analysis of the 12 DNA nanoswitches designed for DENV RNA detection.

Fig. S11. Tuning the loop size of DNA nanoswitch.

Fig. S12. Targets and a gel image of specificity test with Cambodia and Uganda strains of ZIKV.

Fig. S13. Detection of ZIKV RNA in total RNA extracted from human liver cells.

Fig. S14. Gel images of the ZIKV RNA detection in samples mimicking the urine of patients.

Fig. S15. Detection of ZIKV RNA based on pre-amplification with NASBA.

Fig. S16. Portable e-gel system for detection of ZIKV RNA based on pre-amplification with NASBA.

Fig. S17. Detection of a SARS-CoV-2 RNA fragment.

Fig. S18. Detection of SARS-CoV-2 full genome RNA in human saliva.

Fig. S19. Development cycle for DNA nanoswitch based detection of viral RNAs.

Tables

Table S1. A ZIKV RNA target sequence from the literature and its corresponding detector ssDNA (experiments in Fig. 2c).

Table S2. Target sequence and different lengths of detector ssDNA (15, 14, 13, 12, 11, 10 nt) for optimizing the design of nanoswitch (experiments in Fig. S3).

Table S3. The eighteen target sequences and corresponding detector ssDNA oligos for the detection of ZIKV RNA (experiments in Fig. 2e, 2f, 3a, 3b, 3d, 5a and S7, S8, S9).

Table S4. The twelve target sequences and corresponding detector ssDNA oligos for the detection of DENV RNA (experiments in Fig. 3a, S10).

Table S5. Variable oligos for constructing nanoswitches with different loop sizes (experiments in Fig. 3b, 3d).

Table S6. Target sequences and the corresponding detector ssDNA for the ZIKV and DENV multiplexing test (experiments in Fig. 3b).

Table S7. Target sequences and the corresponding detector ssDNA for the ZIKV Cambodia and Uganda specificity test (experiments in Fig. 3d).

Table S8. Amplified region of ZIKV RNA, primers, targets and corresponding detector ssDNA used in NASBA (related experiments in Fig. 5b, S15, S16).

Table S9. DNA template, primers, targets and the corresponding detector ssDNA for SARS-CoV-2 RNA detection.

Table S10. Backbone and basic variable oligos for the construction of nanoswitches and other oligos.

Other supporting information

Matlab code for selecting viral RNA targets for DNA nanoswitch assay (**File 1**)

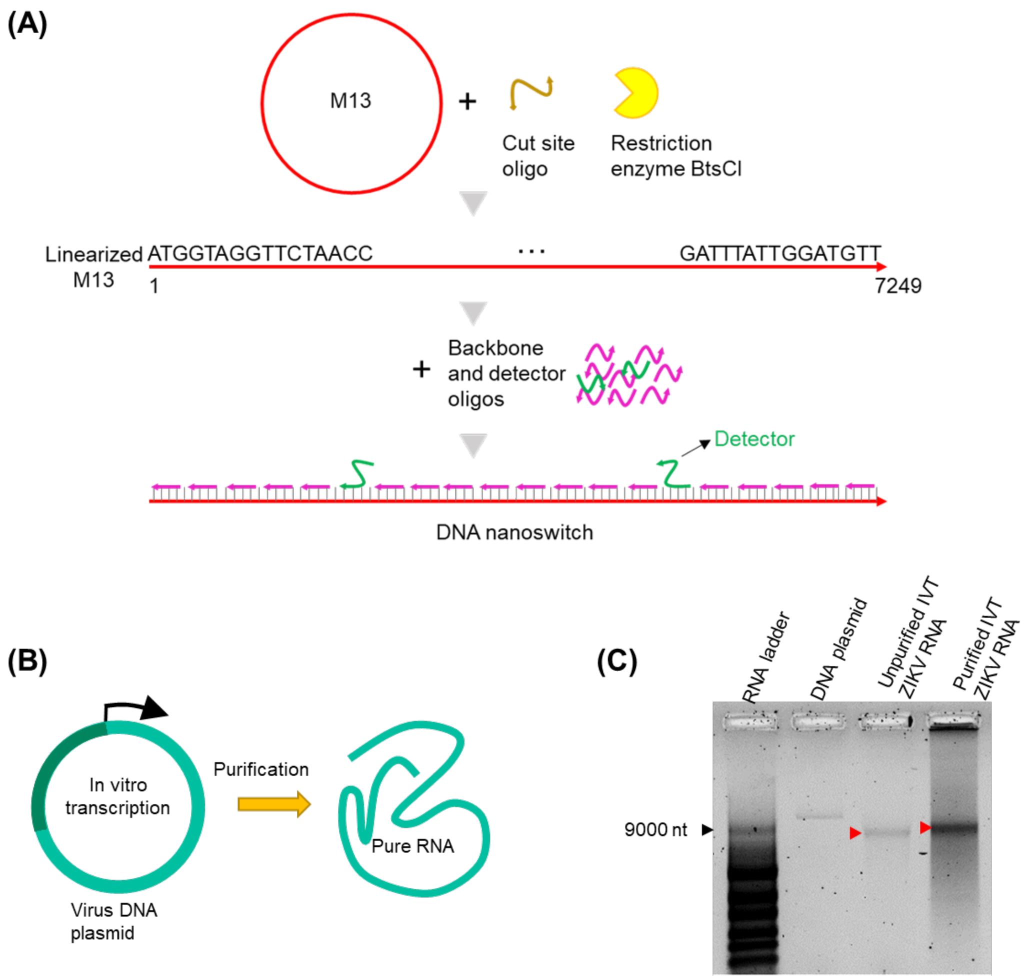

Fig. S1. DNA nanoswitch construction and in vitro transcription (IVT) of viral RNA. (A) Illustration of M13 scaffold linearization and assembly of DNA nanoswitch with backbone oligos and detectors. (**B**) Schematic of *in vitro* transcription reaction. Plasmids containing the full-length infectious cDNA clone of either the ZIKV or DENV genomes were linearized, *in vitro* transcribed, followed by purification of the RNA product. (**C**) Integrity of *in vitro* transcribed (IVT) ZIKV RNA was analyzed by electrophoresis in a native 0.8% agarose/TBE gel. Red arrow indicates the band corresponding to ZIKV RNA. Note: IVT and purification were performed using MEGAscript™ T7 Transcription Kit and MEGAclear™ Transcription Clean-Up Kit from Thermo Fisher Scientific. We followed the protocols of these two kits except that we did not heat the purification column in the elution step of the viral RNA as we noticed that high temperature could result in degradation of the viral RNA.

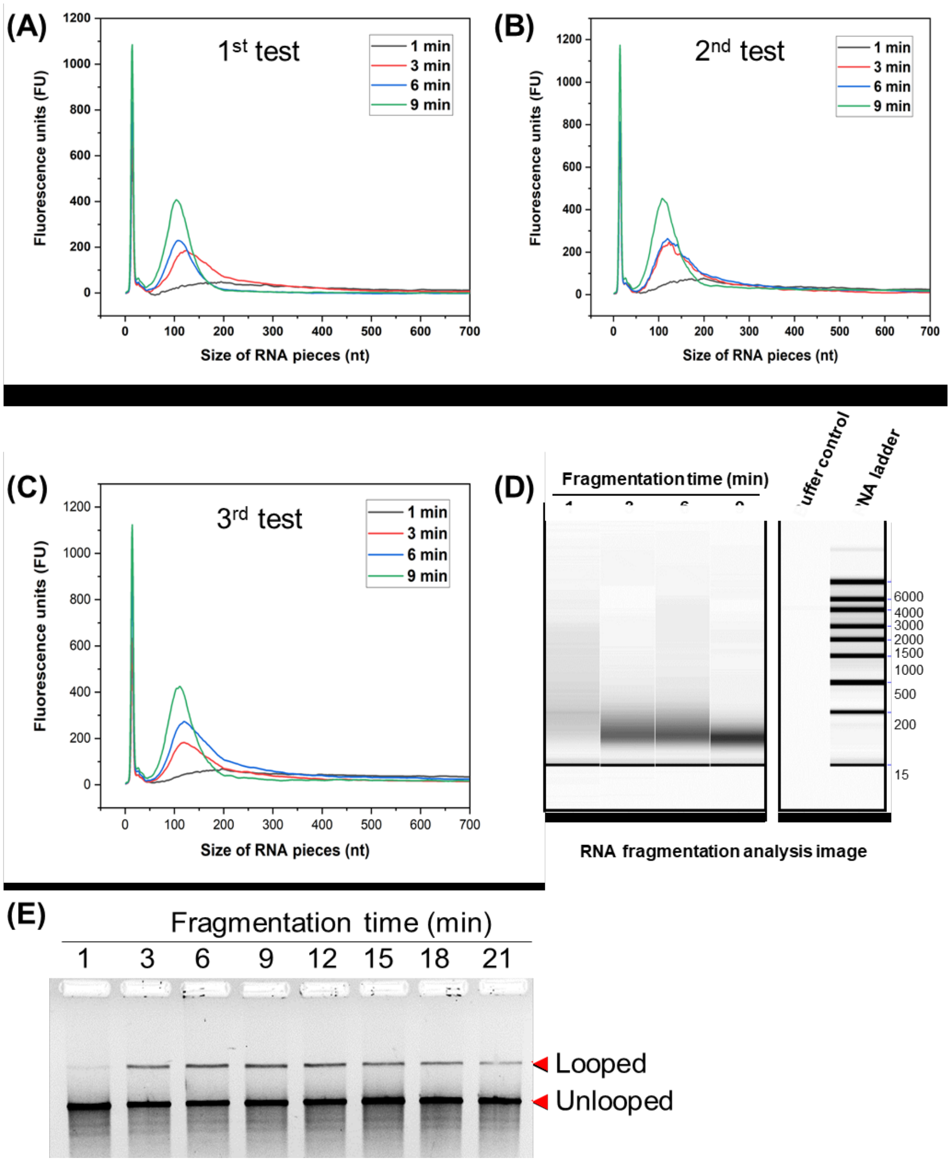

Fig. S2. Fragmentation analysis of ZIKV RNA. (**A**, **B**, **C**) Triplicate results of the ZIKV RNA fragmentation. *In vitro* transcribed ZIKV RNA was fragmented at 94 °C using the RNA fragmentation buffer from New England Biolabs for 1, 3 6 and 9 minutes. (**D**) An example of fragmentation gel image from the RNA fragmentation analyzer showing optimal fragmentation and size following 9 minutes of fragmentation. **(E)** Detection of fragmented ZIKV RNA with different fragmentation times by using 18 nanoswitches mix. Here, 5 ng (~8.5×10^{8} copies) of fragmented *in vitro* transcribed ZIKV RNA was used for each lane.

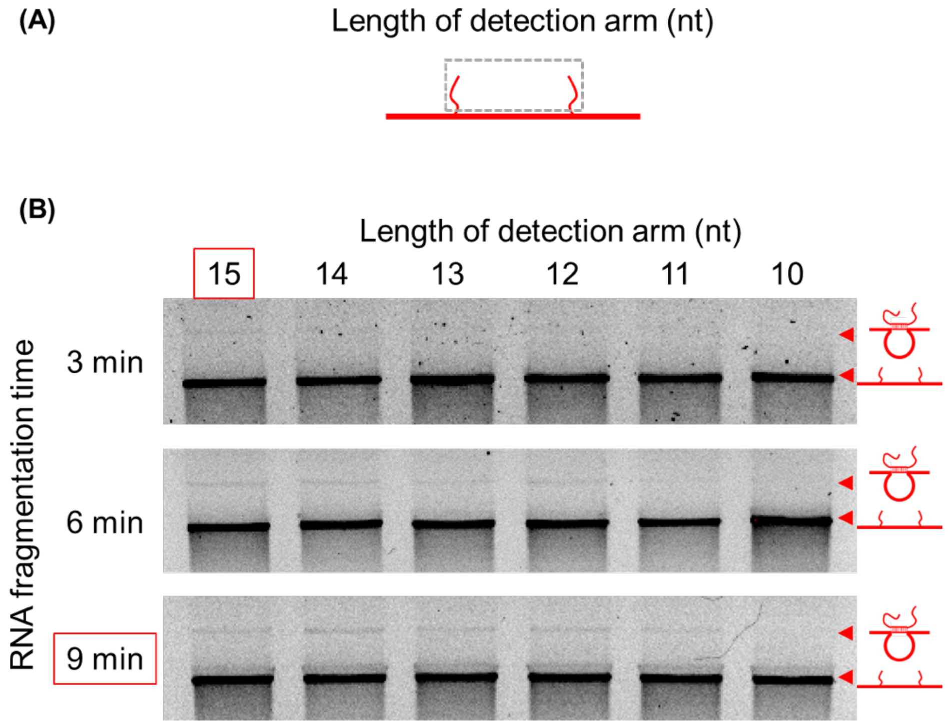

Fig. S3. Optimization of detection arm length. (**A**) Schematic of the DNA nanoswitch. (**B**) Nanoswitches with detector oligonucleotides of different lengths (10-15 nucleotides long) were incubated with *in vitro* transcribed ZIKV RNA that was fragmented at 94 °C with the NEB fragmentation buffer for 3, 6 and 9 minutes. An example 0.8% agarose/TBE gel image showing detection of ZIKV RNA is shown for each fragmentation time. These results revealed optimal detection of ZIKV RNA following 9 minutes of RNA fragmentation and with a nanoswitch containing a 15-nucleotide detector arm length. The nanoswitch used in this experiment is the third nanoswitch in **Table S3**.

Note S1. Choosing the detection targets of viral RNA.

We first determined the target length to be 30 nt based on the detection test results of **Fig. S3**. The ZIKV genome is ~11,000 nucleotides. Within the genome, the RNA can form very stable secondary structures that could inhibit detection by the DNA nanoswitches. We excluded those regions based on the minimum free energy (MFE), a parameter used to indicate the stability of the secondary structures of potential targets. The lower the MFE, the more stable the secondary structure will be. In addition, it is helpful to choose a target sequence with a relatively high GC-content that can enhance the hybridization between the ssDNA detection arms of the nanoswitch and the target RNA. To ensure specificity of our target, we also examined sequence similarity between ZIKV and DENV genome sequences and eliminated those sequences with high alignment scores. When investigating different strains of the same virus the sequence similarity is high and it is effective to pick target regions with as many different nucleotides within the region of interest. The detailed procedure for choosing target sequences is described below and the corresponding tool developed in Matlab and its user instruction can be found in **File S1**.

Step 1: Create the target pool based on the detection region length, GC-content (≥35%) and minimum free energy (≥-2 kcal/mol). The minimum free energy was calculated by using the Matlab function: rnafold(seq).

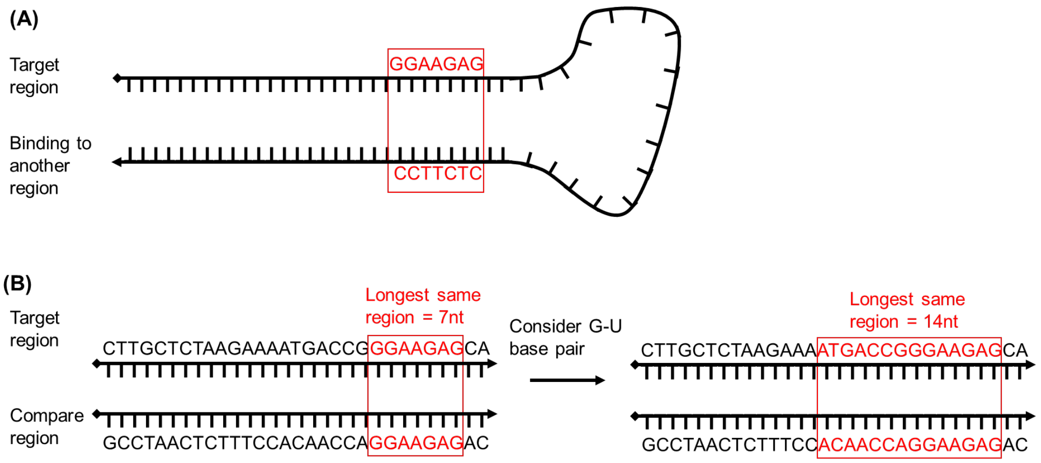

Fig. S4. Considerations for choosing target sequences of viral RNA. (**A**) Schematic of self-binding and formation of a stable secondary structure that should be excluded as a target sequence. (**B**) An example of two comparable targets when G-U base pairing is taken into consideration.

Step 2: Check the potential for strong self-binding within the viral RNA sequence (**Fig. S4A**). The similarity between two target regions was quantified from the alignment score obtained by the Matlab function: nwalign(Seq1,Seq2). Higher alignment score corresponds to higher similarity. When comparing

two regions, the program also computed the number of identical nucleotides and the length of longest adjacent identical nucleotides (**Fig. S4B**). Because G-U base pairing plays an important role in the formation and stabilization of RNA secondary structures, here we also took G-U base pair into account (**Fig. S4B**). Then, we eliminated the pair of targets that have the length of identical adjacent nucleotides longer than 13 nt when G-U base pair is considered.

Step 3: Check the similarity of targets obtained in Step 2 with the DENV RNA sequence (Dengue virus serotype 2, strain Thailand 16681; Genbank accession NC001474) and remove the targets that could result in cross detection with DENV. Here the criteria were that the length of longest adjacent identical nucleotides should be no longer than 15nt and 20nt when G-U base pairing is considered.

Step 4: Check the similarity of targets with the complementary sequence of M13 (p7249), which is used to construct the nanoswitch. This avoids binding of the ssDNA detection arms with the backbone ssDNA (**Fig. S5**). Here the criteria are that the length of longest adjacent identical nucleotides on both halves of the target should be no longer than 6 nt based on our previous research.

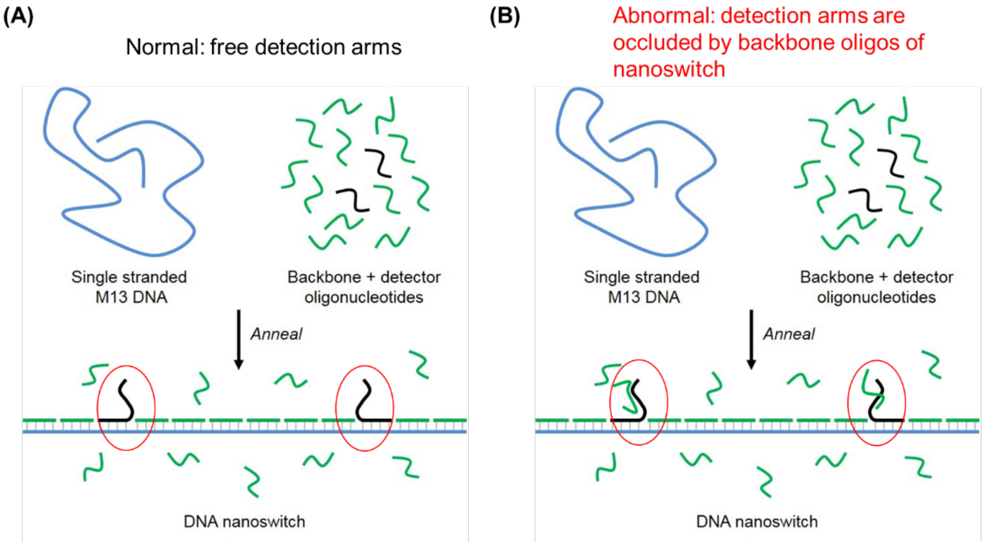

Fig. S5. Schematic showing assembly of DNA nanoswitch and interference by excess backbone oligos. (**A**) Single stranded M13 DNA is annealed with backbone oligonucleotides and detectors specific to the RNA target. Normal assembly results in the detection oligonucleotides have free detection arms. (**B**) In contrast, abnormal assembly of the DNA nanoswitch may result when excess backbone oligonucleotides interact with the detector oligonucleotides and occlude the detection arms thus blocking recognition of target RNA.

Step 5: Pick targets from the final list to ensure the distance between them is longer than 50 nt. The performance of the nanoswitches could be first verified by positive control experiment that uses corresponding ssDNA as the target. The Matlab code with GUI is also provided in the supporting material with instructions for users (see **Fig. S6.** and **File S1**).

Step 6 (Optional): If different strains of the same virus are required to be detected, then the targets should satisfy the requirement that there should be more than 5 mutations between the targeted regions from the two strains. In addition, the position of mutation nucleotides should be near the middle of the detection arm. As the mutation number and position have higher priority than the factors discussed in steps 2-5, the potential targets are screened and picked from the target pool obtained in step 1.

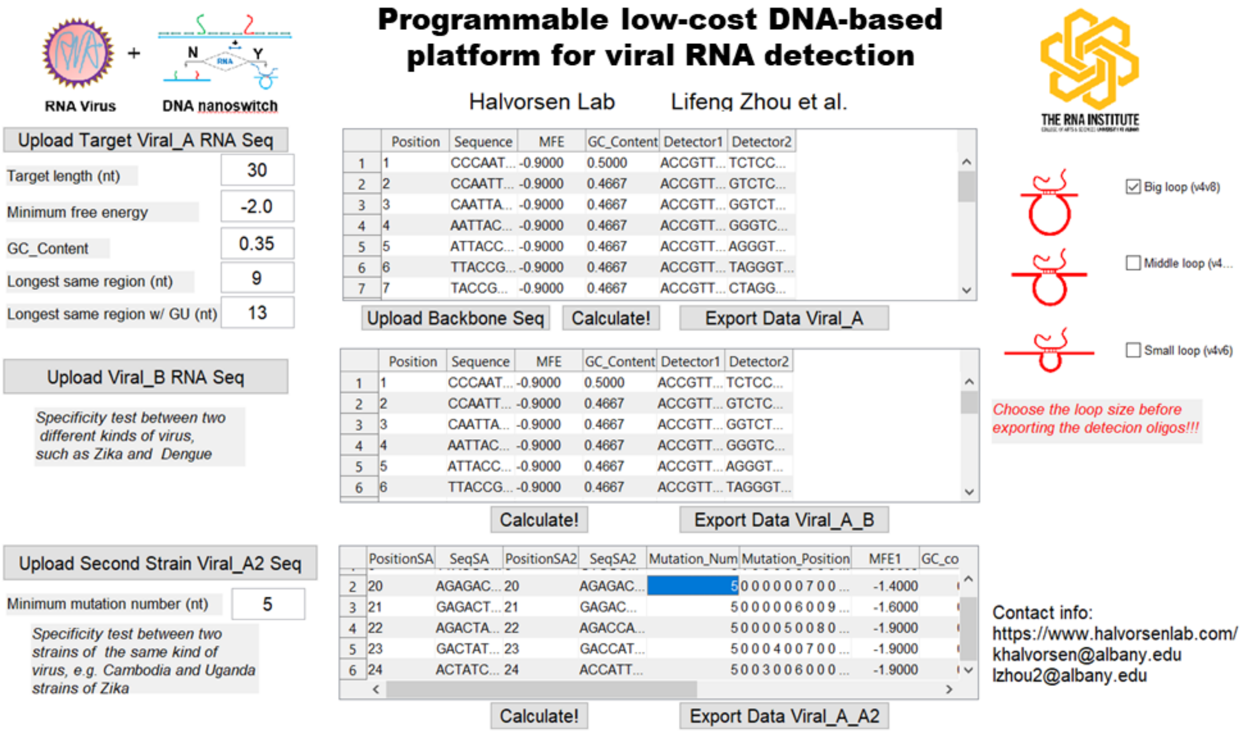

Fig. S6. Graphical user interface (GUI) for obtaining potential viral RNA targets. More requirements could be added to the procedure and the Matlab code can be easily customized to obtain the desired target regions of viral RNAs (See **File S1** for Matlab code and user instructions).

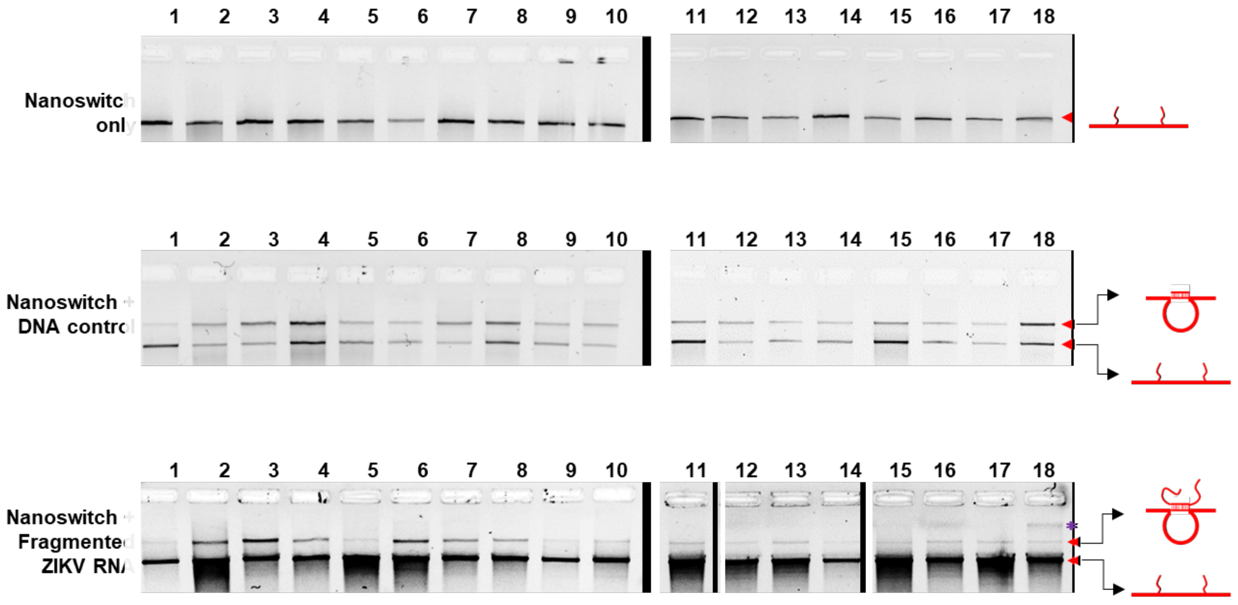

Fig. S7. Analysis of the 18 DNA nanoswitches designed for ZIKV RNA detection. Top panel shows the negative control test of just the different DNA nanoswitches. The middle panel shows the positive control of complementary ssDNA (2 nM) annealed with the corresponding nanoswitch. The bottom panel shows detection of ZIKV RNA by individual DNA nanoswitches. We used 5 ng (~8.5×10^8 copies) of fragmented *in vitro* transcribed ZIKV RNA to test the nanoswitches in 10 µl reaction. * represents dimers formed by DNA nanoswitches.

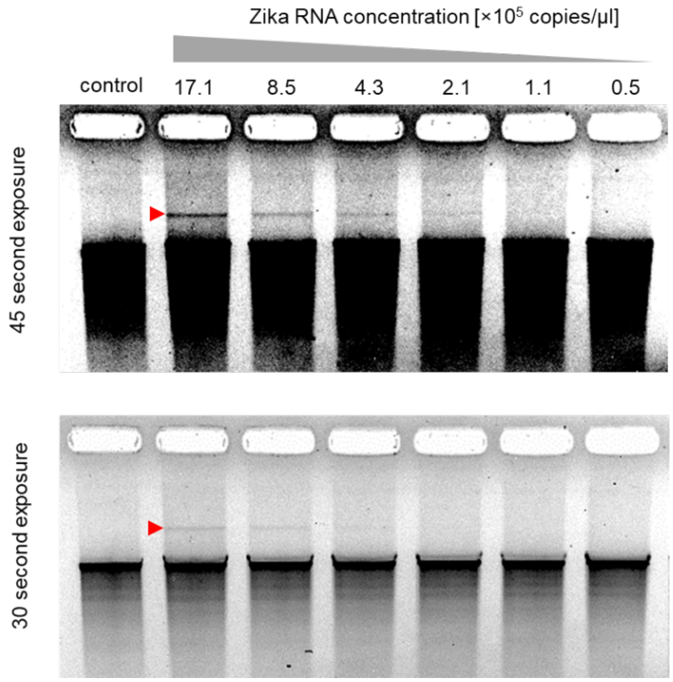

Fig. S8. An example of gel image of the 18 mixed nanoswitches detection sensitivity test. This is the representative gel shown in **Fig. 2F** (45 second exposure is shown at the top and 30 second exposure at the bottom).

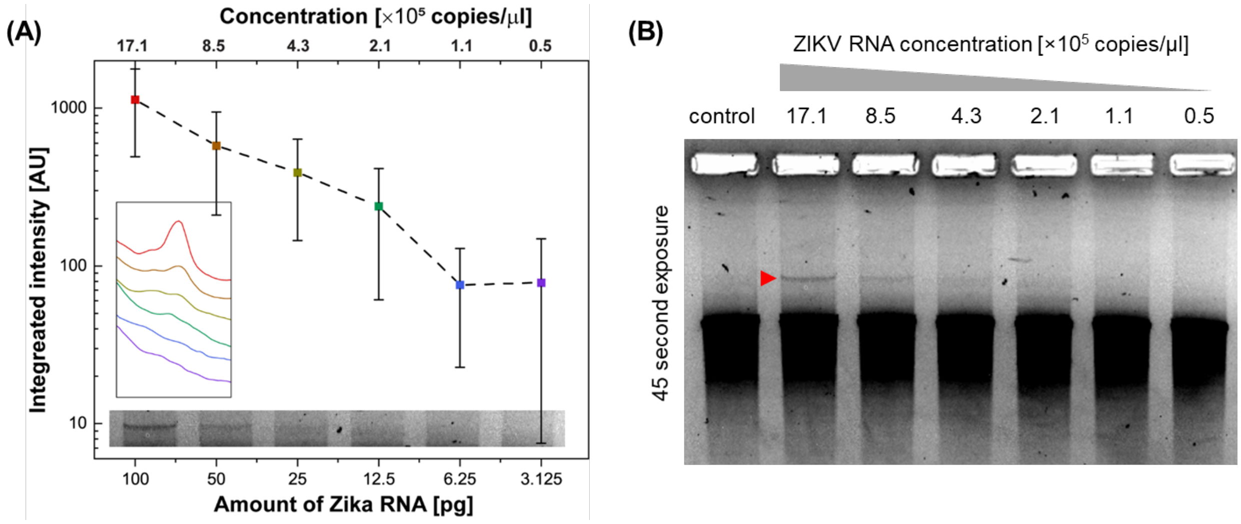

Fig. S9. Detection sensitivity test of single nanoswitch. (**A**) Sensitivity test of a high-performing single nanoswitch (third nanoswitch listed in **Table S3)**. An example of gel image with detection bands is presented as an inset within the graph and the profiles of the detection bands are shown on the left as an inset. (**B**) The entire gel image presented at the bottom of (**A**): A visible band can be seen to at least the $8.5{\times}10^5$ copies/µl (1.4 pM) lane. Experiment was performed in triplicates and error bars represent the standard deviation.

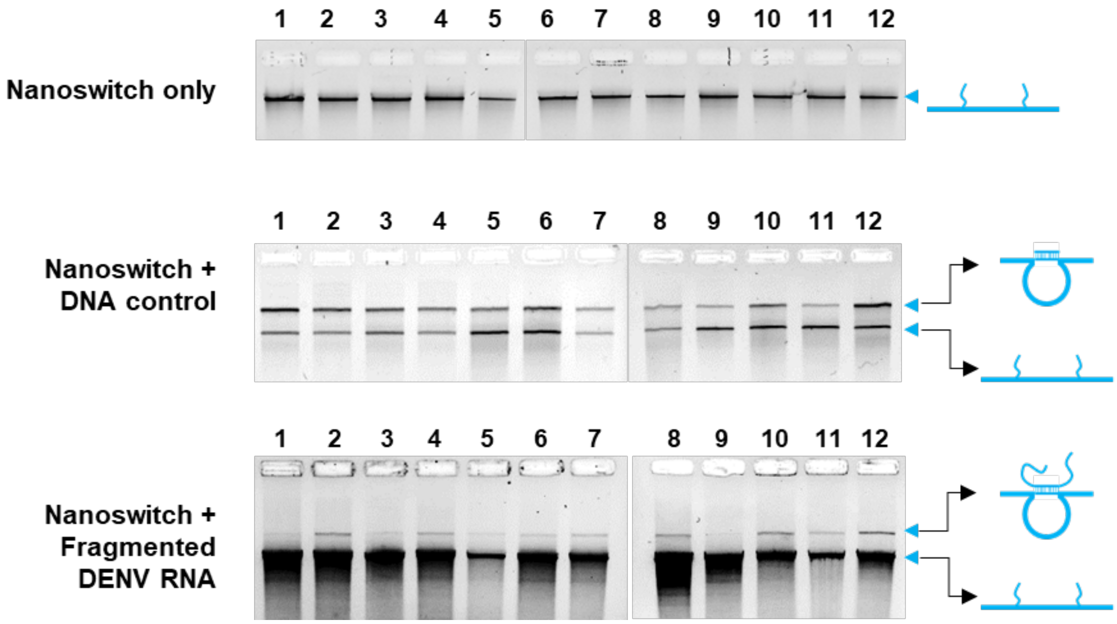

Fig. S10. Analysis of the 12 DNA nanoswitches designed for DENV RNA detection. Top panel shows the negative control test of just the different DNA nanoswitches. The middle panel shows the positive control of complementary ssDNA (2 nM) annealed with the corresponding nanoswitch. The bottom panel shows detection of DENV RNA by individual DNA nanoswitches. We used 10 ng (~1.7×10^9 copies) of fragmented *in vitro* transcribed DENV RNA to test the nanoswitches in 10 µl reaction.

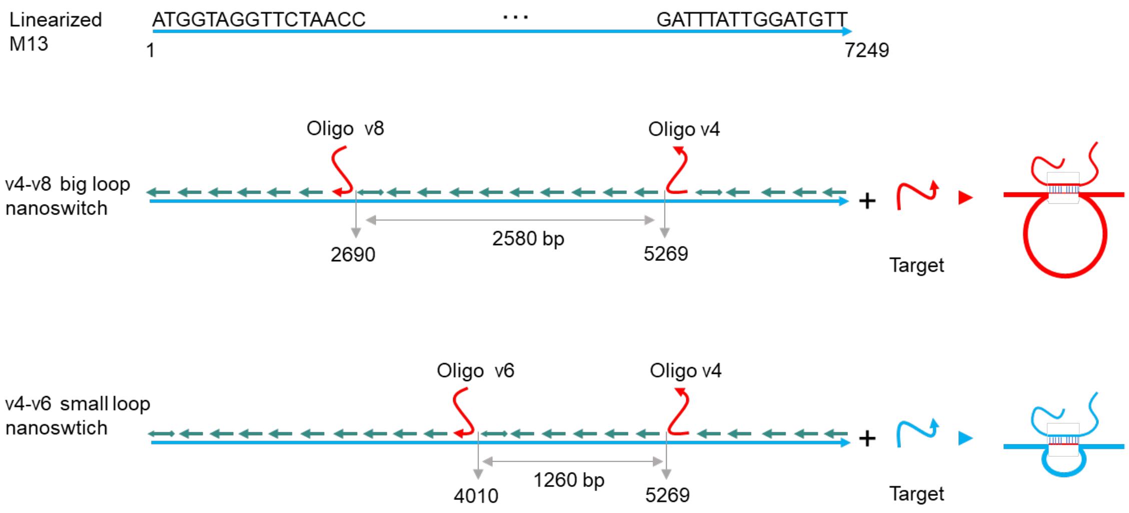

Fig. S11. Tuning the loop size of DNA nanoswitch. The size of v4-v8 loop is about 2580 bp and the size of v4-v6 loop is about 1260 bp. Note in the table of oligos, all detection ssDNA oligos are named with prefix v4- or v8- or v6-.

(A)

Cambodia_1st	AGACTATCATGCTTTTGGGGTTGCTGGGAA
Uganda_1st	AGACCATTATGCTCTTAGGTTTGCTGGGAA
Cambodia_2nd	TTGTTCGGTATGGGTAAAGGGATGCCATTC
Uganda_2nd	CTGTTTGGCATGGGCAAAGGGATGCCATTT
Cambodia_3rd	GCGAAGGTTGAGATAACGCCCAATTCACCA
Uganda_3rd	GCGAAAGTCGAGGTTACGCCTAATTCACCA
Cambodia_4th	GTACCGCAGCGTTCACATTCACTAAGATCC
Uganda_4th	GCACTGCGGCATTCACATTCACCAAGGTCC
Cambodia_5th	CTGCTCTGACAACTTTCATTACCCCAGCCG
Uganda_5th	CCGCATTGACAACTCTCATCACCCCAGCTG

(B)

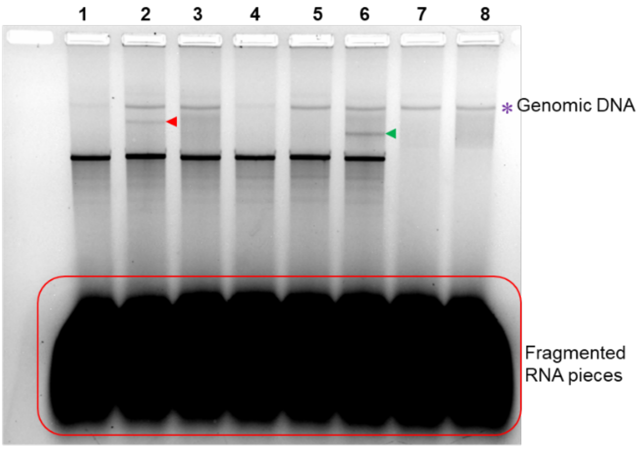

1. Cambodia nanoswitch + fragmented total RNA extracted from mock-infected cells
2. Cambodia nanoswitch + fragmented total RNA extracted from Cambodia strain infected cells
3. Cambodia nanoswitch + fragmented total RNA extracted from Uganda strain infected cells
4. Uganda nanoswitch + fragmented total RNA extracted from mock-infected cells
5. Uganda nanoswitch + fragmented total RNA extracted from Cambodia strain infected cells
6. Uganda nanoswitch + fragmented total RNA extracted from Uganda strain infected cells
7. Fragmented total RNA extracted from Cambodia strain infected cells
8. Fragmented total RNA extracted from Uganda strain infected cells

Fig. S12. Targets and a gel image of specificity test with Cambodia and Uganda strains of ZIKV. (**A**) The five targets for the specificity test of Cambodia and Uganda strains of ZIKV. Strain-specific nucleotides are colored in red. (**B**) A representative gel image from the assay demonstrating nanoswitch specificity for detecting and differentiating between ZIKV Cambodia and Uganda strains used in **Fig. 3C-3D** in the main text. * indicates contaminating cellular DNA left in the total RNA and the area in the red frame at the bottom indicates the unbound fragmented pieces of cellular and viral RNA isolated from mock- and ZIKV-infected Huh7 cells. The oligos of corresponding nanoswitches are listed in **Table S7**.

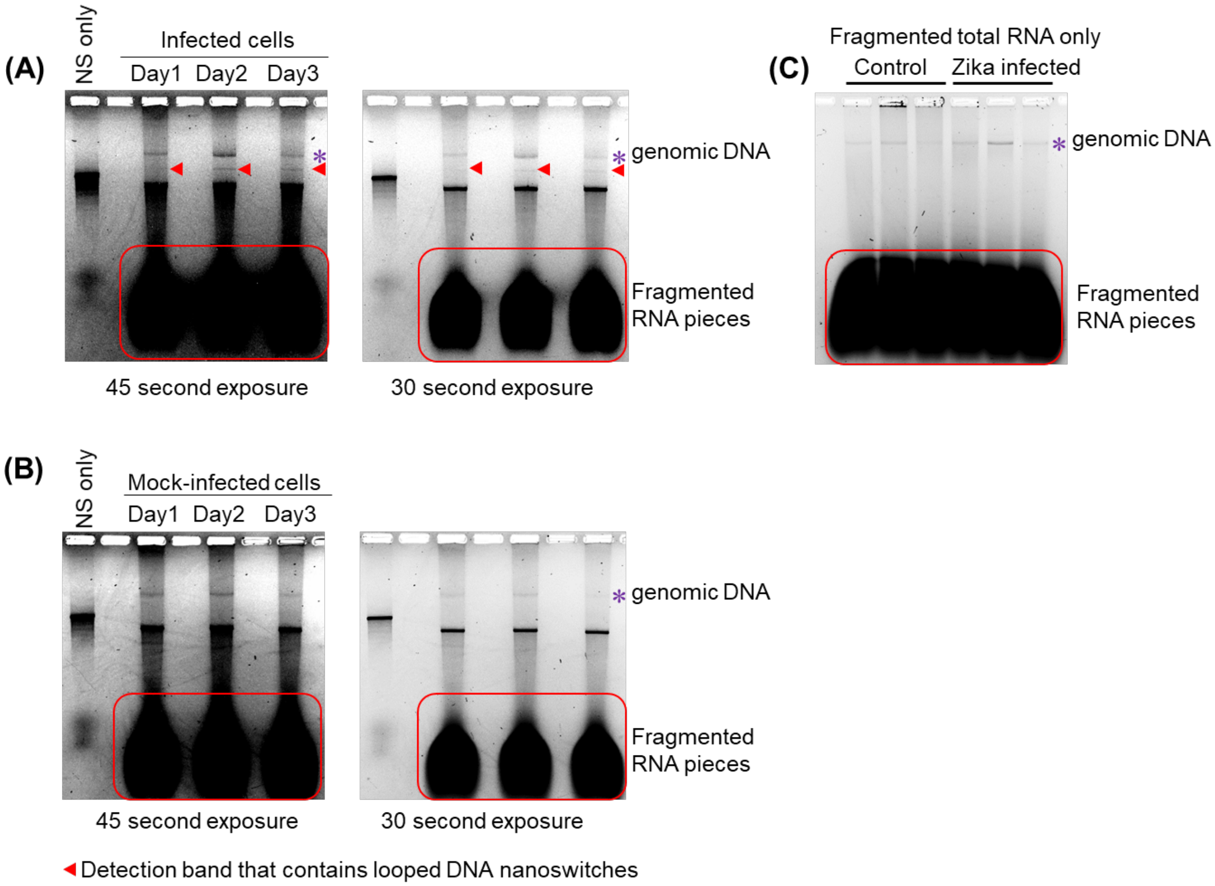

Fig. S13. **Detection of ZIKV RNA in total RNA extracted from human liver cells.** (**A**) Detection of ZIKV RNA in total RNA of infected human liver cells, NS: nanoswitch. (**B**) Control experiment using total RNA from mock-infected human liver cells. (**C**) Fragmented total RNA only. Note: the red arrows indicate the detection bands that contain looped DNA nanoswitches and asterisks indicate the genomic DNA in the total RNA.

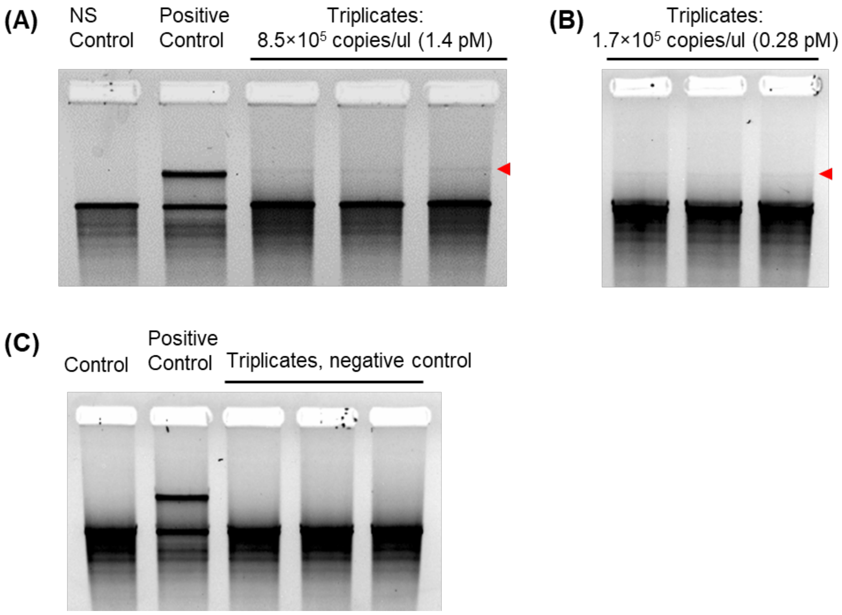

Fig. S14. Gel images of the ZIKV RNA detection in samples mimicking the urine of patients. Triplicate experiments of detecting ZIKV RNA extracted from human urine at (**A**) 8.5×10^5 copies/µl (1.4 pM), (**B**) 1.7×10^5 copies/µl (0.28 pM), and (**C**) negative control. The quantified detection results are presented in **Fig. 5A**.

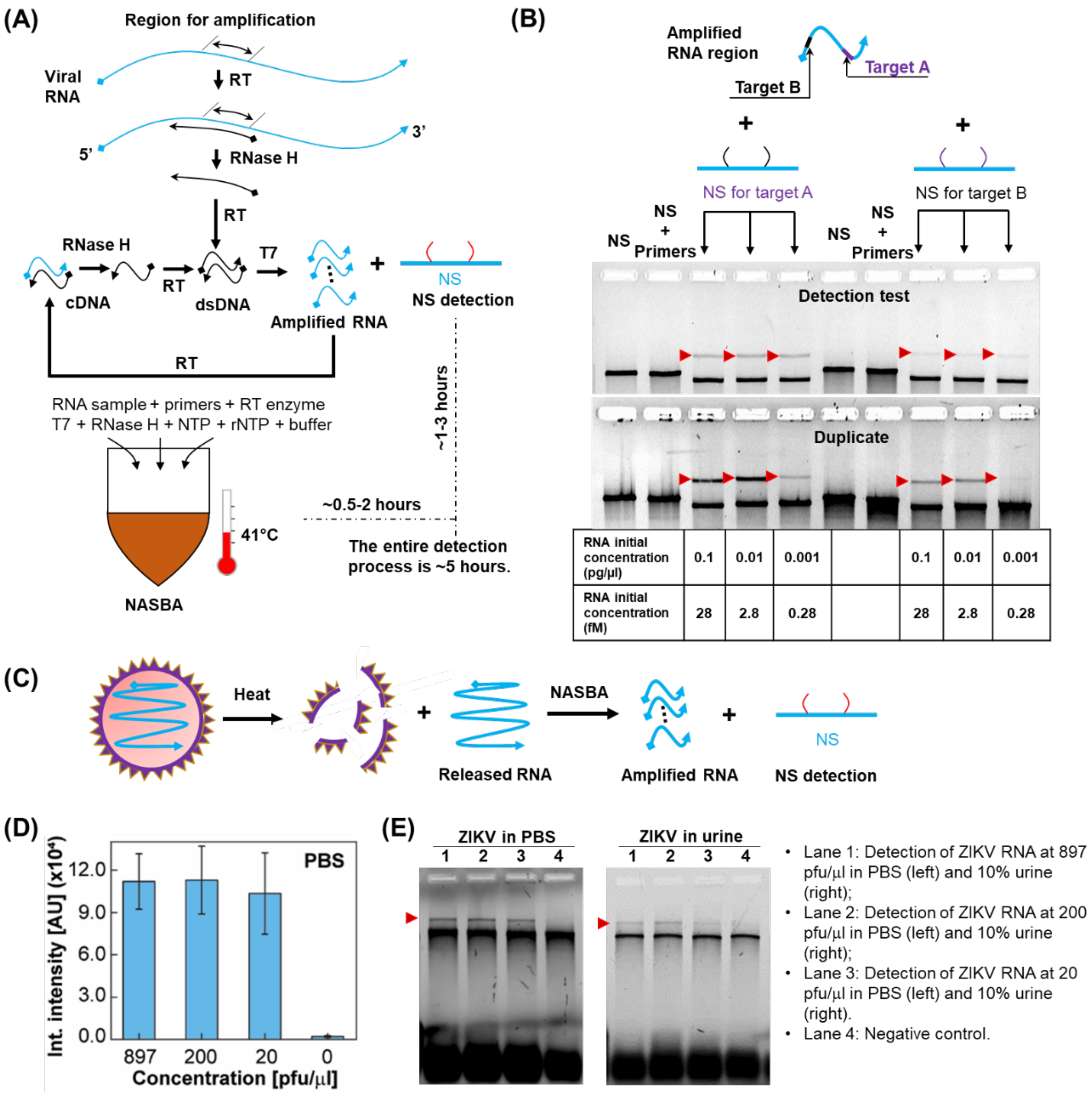

RNA initial concentration (pg/µl)	0.1	0.01	0.001		0.1	0.01	0.001
RNA initial concentration (fM)	28	2.8	0.28		28	2.8	0.28

Fig. S15. Detection of ZIKV RNA based on pre-amplification with NASBA. (**A**) Basic process of Nucleic Acid Sequence Based Amplification (NASBA), RT: reverse transcription. (**B**) Test of detection based on NASBA amplification. Two targets were chosen on the amplified region of the *in vitro* transcribed ZIKV RNA (targets A and B in **Table S8**). (**C**) Schematic of viral RNA detection based on NASBA. (**D**) Positive detection of ZIKV RNA from infectious virus in PBS. (**E**) Example gel images of the ZIKV RNA detection based on NASBA by spiking virus particles into PBS and urine (final concentration is 10%), the nanoswitch used here is the nanoswitch for target A in **Table S8**.

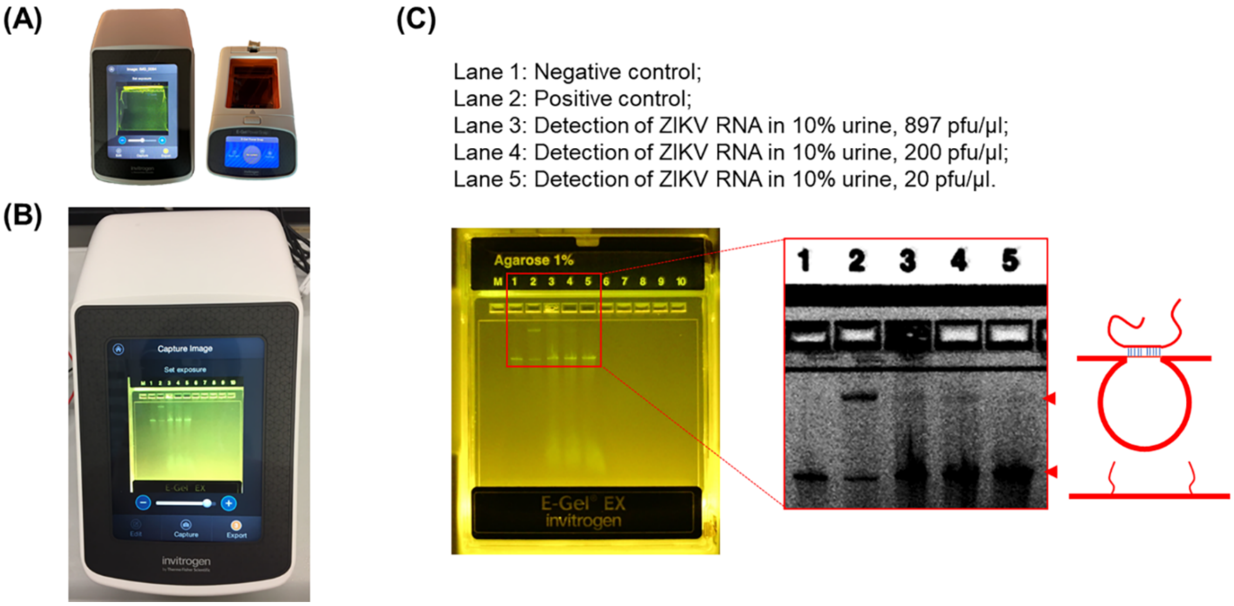

Fig. S16. Portable e-gel system for detection of ZIKV RNA based on pre-amplification with NASBA. (**A**) Commercially available E-gel system, (**B**) Image capture of an E-gel cartridge testing viral nanoswitch detection (run at 48 volts for 1 hour). (**C**) A gel image of the detection of ZIKV RNA based on pre-amplification with NASBA. The concentrations of ZIKV particle in the human urine (10%) are 897, 200 and 20 pfu/µl for lane 3, 4 and 5 respectively. The nanoswitch used here is the one for target A in **Table S8**. Photo Credit: Jibin Abraham Punnoose, The RNA institute.

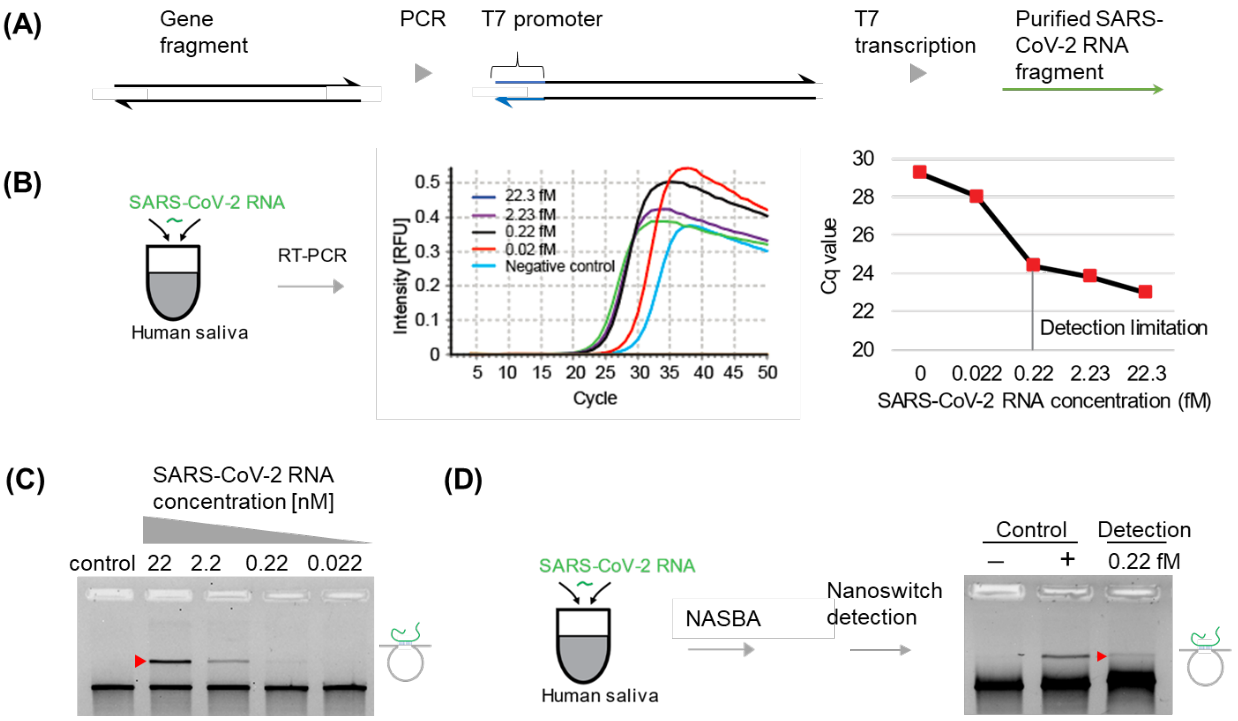

Figure S17. Detection of a SARS-CoV-2 RNA fragment. (**A**) Schematic of producing SARS-CoV-2 RNA fragment. (**B**) RT-PCR detection of SARS-CoV-2 RNA in 10% human saliva. Based on the Cq value shown on the right, the detection limitation of RT-PCR in this scenario is about 0.22 fM. (**C**) Detection test of SARS-CoV-2 RNA with different concentration in buffer. (**D**) Detection of SARS-CoV-2 RNA fragment based on NASBA.

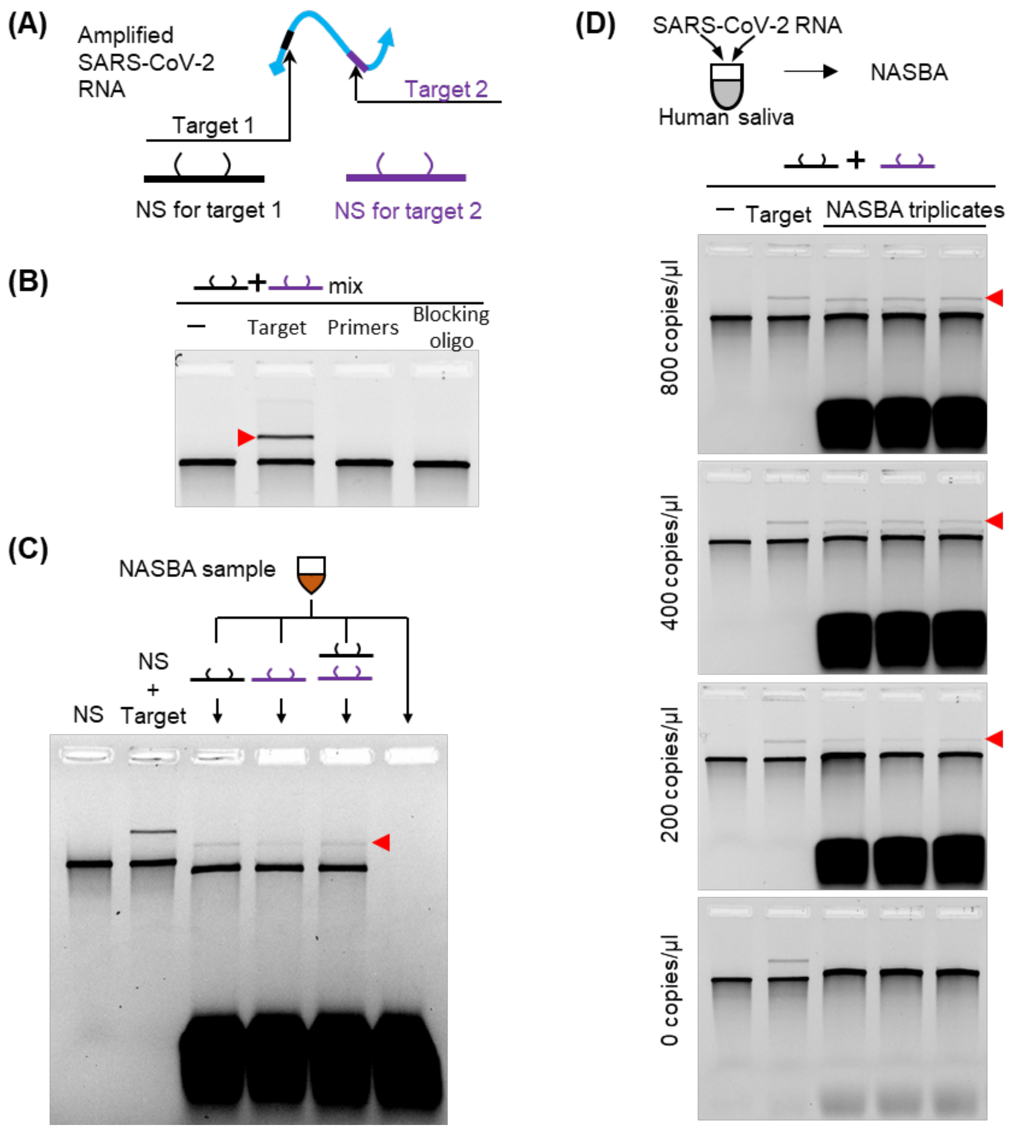

Figure S18. Detection of SARS-CoV-2 full genome RNA in human saliva. (**A**) Sketch of two targets selected on the amplified region by using NASBA. (**B**) Detection of target RNA pieces using a mixture of the two designed nanoswitches. (**C**) Demonstration of the detection ability of the SARS-CoV-2 full genome RNA by using NASBA sample. The gel was run at 75 V for 45 min. (**D**) Detection of different concentrations of SARS-CoV-2 RNA in human saliva. Here, all gels were run at 90 V for 25 min.

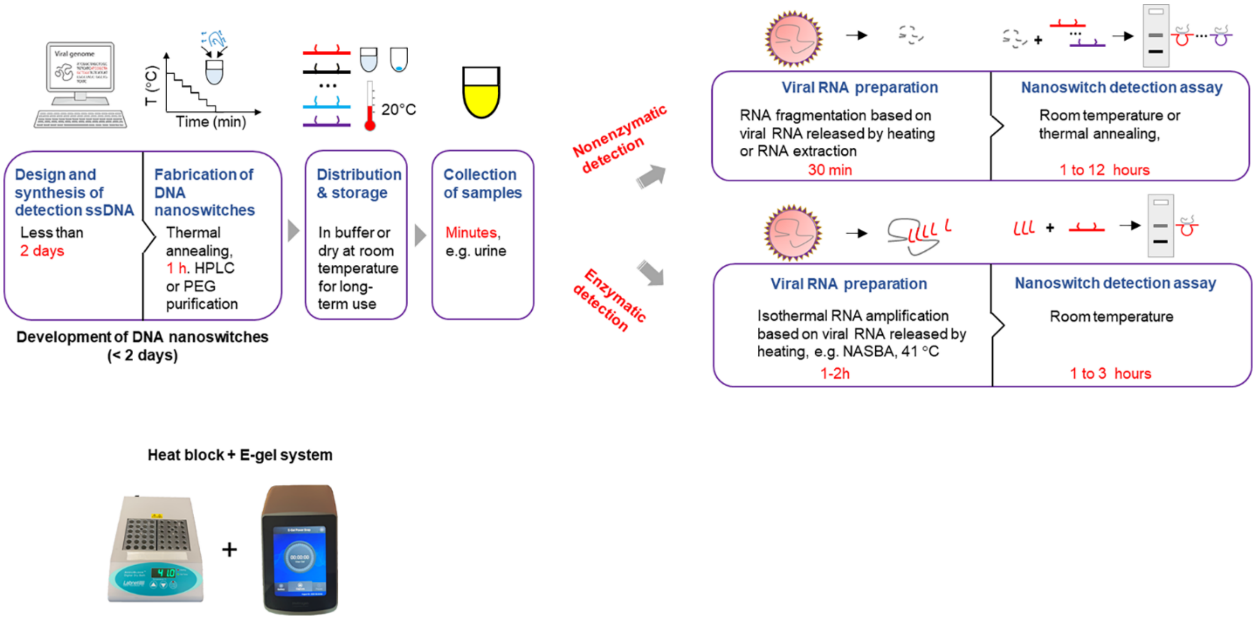

Fig. S19. Development cycle for DNA nanoswitch based detection of viral RNAs. Direct detection can be accomplished in ~1-13 hours and in only 2-5 hours with pre-amplification. Bottom left shows the minimum equipment (heating block and E-gel system, pipettes, tips, and tubes are not shown here) needed for our method. Photo Credit: Jibin Abraham Punnoose, The RNA institute.

Table S1. The ZIKV RNA target sequence and its corresponding detector ssDNA used in the experiment of **Fig. 2C**.

Name	Sequence (5′-3′)	Length
ZIKV_s1_Target	AGCCTACCTTGACAAGCAATCAGACACTCA	30
v4-ZIKV_s1_40-15	ACCGTTGTAGCAATACTTCTTTGATTAGTAATAACATCAC**TGAGTGTCTGATTGC**	55
v8-ZIKV_s1_ 15-40	**TTGTCAAGGTAGGCT**TCAACCGATTGAGGGAGGGAAGGTAAATATTGACGGAAAT	55

Table S2. Target sequence and different lengths of detector ssDNA (15, 14, 13, 12, 11, 10nt) for optimization the design of nanoswitch (**Fig. S3**).

Name	Sequence (5′-3′)	Length
ZIKV_arm length test_Target	AACGCCCAATTCACCAAGAGCCGAAGCCAC	30
v4-ZIKV arm length test 30-15	CAATACTTCTTTGATTAGTAATAACATCAC***GTGGCTTCGGCTCTT***	45
v8-ZIKV arm length test 15-30	***GGTGAATTGGGCGTT***TCAACCGATTGAGGGAGGGAAGGTAAATAT	45
v4-ZIKV arm length test 30-14	CAATACTTCTTTGATTAGTAATAACATCAC***TGGCTTCGGCTCTT***	44
v8-ZIKV arm length test 14-30	***GGTGAATTGGGCGT***TCAACCGATTGAGGGAGGGAAGGTAAATAT	44
v4-ZIKV arm length test 30-13	CAATACTTCTTTGATTAGTAATAACATCAC***GGCTTCGGCTCTT***	43
v8-ZIKV arm length test 13-30	***GGTGAATTGGGCG***TCAACCGATTGAGGGAGGGAAGGTAAATAT	43
v4-ZIKV arm length test 30-12	CAATACTTCTTTGATTAGTAATAACATCAC***GCTTCGGCTCTT***	42
v8-ZIKV arm length test 12-30	***GGTGAATTGGGC***TCAACCGATTGAGGGAGGGAAGGTAAATAT	42
v4-ZIKV arm length test 30-11	CAATACTTCTTTGATTAGTAATAACATCAC***CTTCGGCTCTT***	41
v8-ZIKV arm length test 11-30	***GGTGAATTGGG***TCAACCGATTGAGGGAGGGAAGGTAAATAT	41
v4-ZIKV arm length test 30-10	CAATACTTCTTTGATTAGTAATAACATCAC***TTCGGCTCTT***	40
v8-ZIKV arm length test 10-30	***GGTGAATTGG***TCAACCGATTGAGGGAGGGAAGGTAAATAT	40

Table S3. The eighteen target sequences and corresponding detector ssDNA oligos for the detection of ZIKV RNA (**Fig. 2E, 2F, 3A, 3B, 3D, 5A and S7, S8, S9**).

Note: the position of each target sequence on the ZIKV RNA is shown in the far-right column.

Nanoswitch	Name	Sequence (5′-3′)	Len.	Pos.
1	ZIKV_Target1	GTGTGATGCCACCATGAGCTATGAATGCCC	30	605
	v4-ZIKV T1 40-15	ACCGTTGTAGCAATACTTCTTTGATTAGTAATAACATCAC**GGGCATTCATAGCTC**	55	
	v8-ZIKV T1 15-40	**ATGGTGGCATCACAC**TCAACCGATTGAGGGAGGGAAGGTAAATATTGACGGAAAT	55	
2	ZIKV_Target2	AGTGGACAGAGGCTGGGGAAATGGATGTGG	30	1265
	v4-ZIKV T2 40-15	ACCGTTGTAGCAATACTTCTTTGATTAGTAATAACATCAC**CCACATCCATTTCCC**	55	
	v8-ZIKV T2 15-40	**CAGCCTCTGTCCACT**TCAACCGATTGAGGGAGGGAAGGTAAATATTGACGGAAAT	55	
3	ZIKV_Target3	AACGCCCAATTCACCAAGAGCCGAAGCCAC	30	1484
	v4-ZIKV T3 40-15	ACCGTTGTAGCAATACTTCTTTGATTAGTAATAACATCAC**GTGGCTTCGGCTCTT**	55	
	v8-ZIKV T3 15-40	**GGTGAATTGGGCGTT**TCAACCGATTGAGGGAGGGAAGGTAAATATTGACGGAAAT	55	
4	ZIKV_Target4	AGGGAGTCAAGAAGGAGCAGTTCACACGGC	30	1751
	v4-ZIKV T4 40-15	ACCGTTGTAGCAATACTTCTTTGATTAGTAATAACATCAC**GCCGTGTGAACTGCT**	55	
	v8-ZIKV T4 15-40	**CCTTCTTGACTCCCT**TCAACCGATTGAGGGAGGGAAGGTAAATATTGACGGAAAT	55	
5	ZIKV_Target5	GTACCATCCTGACTCCCCTCGTAGATTGGC	30	2588
	v4-ZIKV T5 40-15	ACCGTTGTAGCAATACTTCTTTGATTAGTAATAACATCAC**GCCAATCTACGAGGG**	55	
	v8-ZIKV T5 15-40	**GAGTCAGGATGGTAC**TCAACCGATTGAGGGAGGGAAGGTAAATATTGACGGAAAT	55	
6	ZIKV_Target6	ACATCATGTGGAGATCAGTAGAAGGGGAGC	30	2683
	v4-ZIKV T6 40-15	ACCGTTGTAGCAATACTTCTTTGATTAGTAATAACATCAC**GCTCCCCTTCTACTG**	55	
	v8-ZIKV T6 15-40	**ATCTCCACATGATGT**TCAACCGATTGAGGGAGGGAAGGTAAATATTGACGGAAAT	55	
7	ZIKV_Target7	GAAGAACGACACATGGAGGCTGAAGAGGGC	30	3104
	v4-ZIKV T7 40-15	ACCGTTGTAGCAATACTTCTTTGATTAGTAATAACATCAC**GCCCTCTTCAGCCTC**	55	
	v8-ZIKV T7 15-40	**CATGTGTCGTTCTTC**TCAACCGATTGAGGGAGGGAAGGTAAATATTGACGGAAAT	55	
8	ZIKV Target 8	CTAATTGGACACCCCGTGAGAGCATGCTGC	30	3835
	v4-ZIKV T8 40-15	ACCGTTGTAGCAATACTTCTTTGATTAGTAATAACATCAC**GCAGCATGCTCTCAC**	55	
	v8-ZIKV T8 15-40	**GGGGTGTCCAATTAG**TCAACCGATTGAGGGAGGGAAGGTAAATATTGACGGAAAT	55	
9	ZIKV_Target9	AAACAGTCCCCGGCTCGATGTGGCACTAGA	30	4430
	v4-ZIKV T9 40-15	ACCGTTGTAGCAATACTTCTTTGATTAGTAATAACATCAC**TCTAGTGCCACATCG**	55	
	v8-ZIKV T9 15-40	**AGCCGGGGACTGTTT**TCAACCGATTGAGGGAGGGAAGGTAAATATTGACGGAAAT	55	
10	ZIKV_Target10	CCCGGAGAGAGAGCGAGGAACATCCAGACT	30	4917
	v4-ZIKV T10 40-15	ACCGTTGTAGCAATACTTCTTTGATTAGTAATAACATCAC**AGTCTGGATGTTCCT**	55	
	v8-ZIKV T10 15-40	**CGCTCTCTCTCCGGG**TCAACCGATTGAGGGAGGGAAGGTAAATATTGACGGAAAT	55	
11	ZIKV_Target11	GGACTACCCAGCAGGAACTTCAGGATCTCC	30	4997
	v4-ZIKV T11 40-15	ACCGTTGTAGCAATACTTCTTTGATTAGTAATAACATCAC**GGAGATCCTGAAGTT**	55	

	v8-ZIKV T11 15-40	**CCTGCTGGGTAGTCC**TCAACCGATTGAGGGAGGGAAGGTAAATATTGACGGAAAT	55	
12	ZIKV_Target12	GTGACGCATTCCCGGACTCCAACTCACCAA	30	5581
	v4-ZIKV T12 40-15	ACCGTTGTAGCAATACTTCTTTGATTAGTAATAACATCAC**TTGGTGAGTTGGAGT**	55	
	v8-ZIKV T12 15-40	**CCGGGAATGCGTCAC**TCAACCGATTGAGGGAGGGAAGGTAAATATTGACGGAAAT	55	
13	ZIKV_Target13	GAGTTCCAGAAAACAAAACATCAAGAGTGG	30	5793
	v4-ZIKV T13 40-15	ACCGTTGTAGCAATACTTCTTTGATTAGTAATAACATCAC**CCACTCTTGATGTTT**	55	
	v8-ZIKV T13 15-40	**TGTTTTCTGGAACTC**TCAACCGATTGAGGGAGGGAAGGTAAATATTGACGGAAAT	55	
14	ZIKV_Target14	CATCTAATGGGAAGGAGAGAGGAGGGGGCA	30	6957
	v4-ZIKV T14 40-15	ACCGTTGTAGCAATACTTCTTTGATTAGTAATAACATCAC**TGCCCCCTCCTCTCT**	55	
	v8-ZIKV T14 15-40	**CCTTCCCATTAGATG**TCAACCGATTGAGGGAGGGAAGGTAAATATTGACGGAAAT	55	
15	ZIKV_Target15	CACAGGAATAGCCATGACCGACACCACACC	30	8684
	v4-ZIKV T15 40-15	ACCGTTGTAGCAATACTTCTTTGATTAGTAATAACATCAC**GGTGTGGTGTCGGTC**	55	
	v8-ZIKV T15 15-40	**ATGGCTATTCCTGTG**TCAACCGATTGAGGGAGGGAAGGTAAATATTGACGGAAAT	55	
16	ZIKV_Target16	GGATGGGGAGAGAGAATTCAGGAGGTGGTG	30	9160
	v4-ZIKV T16 40-15	ACCGTTGTAGCAATACTTCTTTGATTAGTAATAACATCAC**CACCACCTCCTGAAT**	55	
	v8-ZIKV T16 15-40	**TCTCTCTCCCCATCC**TCAACCGATTGAGGGAGGGAAGGTAAATATTGACGGAAAT	55	
17	ZIKV_Target17	GAGGAAGTTCTAGAGATGCAAGACTTGTGG	30	9549
	v4-ZIKV T17 40-15	ACCGTTGTAGCAATACTTCTTTGATTAGTAATAACATCAC**CCACAAGTCTTGCAT**	55	
	v8-ZIKV T17 15-40	**CTCTAGAACTTCCTC**TCAACCGATTGAGGGAGGGAAGGTAAATATTGACGGAAAT	55	
18	ZIKV_Target18	CTGAGTCAAAAAACCCCACGCGCTTGGAGG	30	10543
	v4-ZIKV T18 40-15	ACCGTTGTAGCAATACTTCTTTGATTAGTAATAACATCAC**CCTCCAAGCGCGTGG**	55	
	v8-ZIKV T18 15-40	**GGTTTTTTGACTCAG**TCAACCGATTGAGGGAGGGAAGGTAAATATTGACGGAAAT	55	

Table S4. The twelve target sequences and corresponding detector ssDNA oligos for the detection of DENV RNA (**Fig. 3A, S10**).

Note: the position of each target sequence on the DENV RNA is shown in the far-right column.

Nanoswitch	Name	Sequence (5′-3′)	Len.	Pos.
1	DENV Target 1	GTGACTGAGGACTGCGGAAATAGAGGACCC	30	2823
	v4-DENV T1 40-15	ACCGTTGTAGCAATACTTCTTTGATTAGTAATAACATCAC**GGGTCCTCTATTTCC**	55	
	v8-DENV T1 15-40	**GCAGTCCTCAGTCAC**TCAACCGATTGAGGGAGGGAAGGTAAATATTGACGGAAAT	55	
2	DENV Target 2	CTCTCCTCCCAGAGCACTATACCAGAGACC	30	3280
	v4-DENV T2 40-15	ACCGTTGTAGCAATACTTCTTTGATTAGTAATAACATCAC**GGTCTCTGGTATAGT**	55	
	v8-DENV T2 15-40	**GCTCTGGGAGGAGAG**TCAACCGATTGAGGGAGGGAAGGTAAATATTGACGGAAAT	55	
3	DENV Target 3	TGCTCACTGGACGATCGGCCGATTTGGAAC	30	3805
	v4-DENV T3 40-15	ACCGTTGTAGCAATACTTCTTTGATTAGTAATAACATCAC**GTTCCAAATCGGCCG**	55	

	v8-DENV T3 15-40	**ATCGTCCAGTGAGCA**TCAACCGATTGAGGGAGGGAAGGTAAATATTG ACGGAAAT	55	
4	DENV Target 4	GGCCAGCACTCCAAGCAAAAGCATCCAGAG	30	4259
	v4-DENV T4 40-15	ACCGTTGTAGCAATACTTCTTTGATTAGTAATAACATCAC**CTCTGGA TGCTTTTG**	55	
	v8-DENV T4 15-40	**CTTGGAGTGCTGGCC**TCAACCGATTGAGGGAGGGAAGGTAAATATTG ACGGAAAT	55	
5	DENV Target 5	CACACCAGAAGGGAAAGTAGTGGACCTCGG	30	5488
	v4-DENV T5 40-15	ACCGTTGTAGCAATACTTCTTTGATTAGTAATAACATCAC**CCGAGGT CCACTACT**	55	
	v8-DENV T5 15-40	**TTCCCTTCTGGTGTG**TCAACCGATTGAGGGAGGGAAGGTAAATATTG ACGGAAAT	55	
6	DENV Target 6	AAGCCACTTACGAGCCGGATGTTGACCTCG	30	6432
	v4-DENV T6 40-15	ACCGTTGTAGCAATACTTCTTTGATTAGTAATAACATCAC**CGAGGTC AACATCCG**	55	
	v8-DENV T6 15-40	**GCTCGTAAGTGGCTT**TCAACCGATTGAGGGAGGGAAGGTAAATATTG ACGGAAAT	55	
7	DENV Target 7	GCATGGCGTAGTGGACTAGCGGTTAGAGGA	30	7190
	v4-DENV T7 40-15	ACCGTTGTAGCAATACTTCTTTGATTAGTAATAACATCAC**TCCTCTA ACCGCTAG**	55	
	v8-DENV T7 15-40	**TCCACTACGCCATGC**TCAACCGATTGAGGGAGGGAAGGTAAATATTG ACGGAAAT	55	
8	DENV Target 8	CAAGCTACAGCTCAAAGGAATGTCATACTC	30	7782
	v4-DENV T8 40-15	ACCGTTGTAGCAATACTTCTTTGATTAGTAATAACATCAC**GAGTATG ACATTCCT**	55	
	v8-DENV T8 15-40	**TTGAGCTGTAGCTTG**TCAACCGATTGAGGGAGGGAAGGTAAATATTG ACGGAAAT	55	
9	DENV Target 9	GACCCATTTCCTCAGAGCAATGCACCAATC	30	8315
	v4-DENV T9 40-15	ACCGTTGTAGCAATACTTCTTTGATTAGTAATAACATCAC**GATTGGT GCATTGCT**	55	
	v8-DENV T9 15-40	**CTGAGGAAATGGGTC**TCAACCGATTGAGGGAGGGAAGGTAAATATTG ACGGAAAT	55	
10	DENV Target 13	GAAGGCAAGAAACGCACTGGACAACTTAGC	30	8653
	v4-DENV T10 40-15	ACCGTTGTAGCAATACTTCTTTGATTAGTAATAACATCAC**GCTAAGT TGTCCAGT**	55	
	v8-DENV T10 15-40	**GCGTTTCTTGCCTTC**TCAACCGATTGAGGGAGGGAAGGTAAATATTG ACGGAAAT	55	
11	DENV Target 11	AGAACCCAAGAACCGAAAGAAGGCACGAAG	30	9313
	v4-DENV T11 40-15	ACCGTTGTAGCAATACTTCTTTGATTAGTAATAACATCAC**CTTCGTG CCTTCTTT**	55	
	v8-DENV T11 15-40	**CGGTTCTTGGGTTCT**TCAACCGATTGAGGGAGGGAAGGTAAATATTG ACGGAAAT	55	
12	DENV Target 12	AGACCAACACCAAGAGGCACAGTAATGGAC	30	10481
	v4-DENV T12 40-15	ACCGTTGTAGCAATACTTCTTTGATTAGTAATAACATCAC**GTCCATT ACTGTGCC**	55	
	v8-DENV T12 15-40	**TCTTGGTGTTGGTCT**TCAACCGATTGAGGGAGGGAAGGTAAATATTG ACGGAAAT	55	

Table S5. Variable oligos for constructing nanoswitches with different loop sizes used in **Fig. 3B, 3D**.

	Name	Sequence (5′-3′)	Length
**For v4-v8 loop nanoswitch**	v4 oligo	Oligos with prefix ‘v4-’	
	v8 oligo	Oligos with prefix ‘v8-’	
	Var 4 filler	TCTGTCCATCACGCAAATTA	20
	Var 8 filler	TATTCATTAAAGGTGAATTA	20
	v4 oligo	Oligos with prefix ‘v4-’	

**For v4-v6 loop nanoswitch**	v8 oligo	Oligos with prefix ‘v6-’	
	Var 4 filler	TCTGTCCATCACGCAAATTA	20
	Var 6 filler	TCGCAAGACAAAGAACGCGA	20
**For v4-v7 loop nanoswitch**	v4 oligo	Oligos with prefix ‘v4-’	
	v7 oligo	Oligos with prefix ‘v7-’	20
	Var 4 filler	TCTGTCCATCACGCAAATTA	20
	Var 7 filler	TCGCAAGACAAAGAACGCGA	

Table S6. Target sequences and the corresponding detector ssDNA for the ZIKV and DENV multiplexing test (**Fig. 3B**).

Name	Sequence (5′-3′)	Len.
ZIKV_Target3	AACGCCCAATTCACCAAGAGCCGAAGCCAC	30
v4-ZIKV T3 40-15	ACCGTTGTAGCAATACTTCTTTGATTAGTAATAACATCAC**GTGGCTTCGGCTCTT**	55
v8-ZIKV T3 15-40	**GGTGAATTGGGCGTT**TCAACCGATTGAGGGAGGGAAGGTAAATATTGACGGAAAT	55
DENV_Target 10	GCATGGCGTAGTGGACTAGCGGTTAGAGGA	30
v4-DENV T10 40-15	ACCGTTGTAGCAATACTTCTTTGATTAGTAATAACATCAC**TCCTCTAACCGCTAG**	55
v6-DENV T10 15-40	**TCCACTACGCCATGC**TGGGTTATATAACTATATGTAAATGCTGATGCAAATCCAA	55

Table S7. Target sequences and the corresponding detection arm ssDNA for the ZIKV Cambodia and Uganda specificity test (**Fig. 3D**).

Name	Sequence (5′-3′)	Len.
Cambodia_1st	AGAC**T**AT**C**ATGCT**T**TT**G**GG**G**TTGCTGGGAA	30
v4-Cambodia_1st_ 40_15	ACCGTTGTAGCAATACTTCTTTGATTAGTAATAACATCAC**TTCCCAGCAACCCCA**	55
v8-Cambodia_1st_15_40	**AAAGCATGATAGTCT**TCAACCGATTGAGGGAGGGAAGGTAAATATTGACGGAAAT	55
Cambodia_2nd	**T**TGTT**C**GG**T**ATGGG**T**AAAGGGATGCCATT**C**	30
v4-Cambodia_2nd_ 40_15	ACCGTTGTAGCAATACTTCTTTGATTAGTAATAACATCAC**GAATGGCATCCCTTT**	55
v8-Cambodia_2nd_ 15_40	**ACCCATACCGAACAA**TCAACCGATTGAGGGAGGGAAGGTAAATATTGACGGAAAT	55
Cambodia_3rd	GCGAA**G**GT**T**GAG**A**T**A**ACGCC**C**AATTCACCA	30
v4-Cambodia_3rd_ 40_15	ACCGTTGTAGCAATACTTCTTTGATTAGTAATAACATCAC**TGGTGAATTGGGCGT**	55
v8-Cambodia_3rd_ 15_40	**TATCTCAACCTTCGC**TCAACCGATTGAGGGAGGGAAGGTAAATATTGACGGAAAT	55
Cambodia_4th	G**T**AC**C**GC**A**GC**G**TTCACATTCAC**T**AAG**A**TCC	30
v4-Cambodia_4th_ 40_15	ACCGTTGTAGCAATACTTCTTTGATTAGTAATAACATCAC**GGATCTTAGTGAATG**	55
v8-Cambodia_4th_ 15_40	**TGAACGCTGCGGTAC**TCAACCGATTGAGGGAGGGAAGGTAAATATTGACGGAAAT	55
Cambodia_5th	C**T**GC**TC**TGACAACT**T**TCAT**T**ACCCCAGC**C**G	30
v4-Cambodia_5th_ 40_15	ACCGTTGTAGCAATACTTCTTTGATTAGTAATAACATCAC**CGGCTGGGGTAATGA**	55

v8-Cambodia_5th_ 15_40	**AAGTTGTCAGAGCAG**TCAACCGATTGAGGGAGGGAAGGTAAATATTGACGGAAAT	55
Uganda_1st	AGAC**C**AT**T**ATGCT**C**TT**A**GG**T**TTGCTGGGAA	30
v4-Uganda_1st_40_15	ACCGTTGTAGCAATACTTCTTTGATTAGTAATAACATCAC**TTCCCAGCAAACCTA**	55
v7-Uganda_1st_15_40	**AGAGCATAATGGTCT**GTTTTAGCGAACCTCCCGACTTGCGGGAGGTTTTGAAGCC	55
Uganda_2nd	**C**TGTT**T**GG**C**ATGGG**C**AAAGGGATGCCATT**T**	30
v4-Uganda_2nd_40_15	ACCGTTGTAGCAATACTTCTTTGATTAGTAATAACATCAC**AAATGGCATCCCTTT**	55
v7-Uganda_2nd_15_40	**GCCCATGCCAAACAG**GTTTTAGCGAACCTCCCGACTTGCGGGAGGTTTTGAAGCC	55
Uganda_3rd	GCGAA**A**GT**C**GAG**G**T**T**ACGCC**T**AATTCACCA	30
v4-Uganda_3rd_40_15	ACCGTTGTAGCAATACTTCTTTGATTAGTAATAACATCAC**TGGTGAATTAGGCGT**	55
v7-Uganda_3rd_15_40	**AACCTCGACTTTCGC**GTTTTAGCGAACCTCCCGACTTGCGGGAGGTTTTGAAGCC	55
Uganda_4th	G**C**AC**T**GC**G**GC**A**TTCACATTCAC**C**AAG**G**TCC	30
v4-Uganda_4th_40_15	ACCGTTGTAGCAATACTTCTTTGATTAGTAATAACATCAC**GGACCTTGGTGAATG**	55
v7-Uganda_4th_15_40	**TGAATGCCGCAGTGC**GTTTTAGCGAACCTCCCGACTTGCGGGAGGTTTTGAAGCC	55
Uganda_5th	C**C**GC**AT**TGACAACT**C**TCAT**C**ACCCCAGC**T**G	30
v4-Uganda_5th_40_15	ACCGTTGTAGCAATACTTCTTTGATTAGTAATAACATCAC**CAGCTGGGGTGATGA**	55
v7-Uganda_5th_15_40	**GAGTTGTCAATGCGG**GTTTTAGCGAACCTCCCGACTTGCGGGAGGTTTTGAAGCC	55

Table S8. Amplified region of ZIKV RNA, primers, targets and corresponding detector ssDNA used in NASBA related experiments in **Fig. 5B, S15, S16**..

The forward primer has a T7 promoter: AATTCTAATACGACTCACTATAGGGAGAAGG.

Name	Sequence (5′-3′)	Len.
Amplified region on ZIKV RNA (1394-1560)	**AATGCTGTCAGTTCATGGCTCCCA**GCACAGTGGGATGATCGTTAATGATACAGGACATGAAACTGATGAGAATAGAGCGAAGGTTGAGATAACGCCCAATTCACCAAGAGCCGAAGCCACCCTGGGGGGTTTTGGAAGCCTAGGACT**TGATTGTGAACCGAGGACAG**	167
ZIKV NASBA_Reverse primer	CTGTCCTCGGTTCACAATCA	20
ZIKV NASBA_Forward primer	**AATTCTAATACGACTCACTATAGGGAGAAGG**AATGCTGTCAGTTCATGGCTCCCA	55
ZIKV_NASBA_Target A	AACGCCCAATTCACCAAGAGCCGAAGCCAC	30
v4-ZIKV NASBA_Target A 40-15	ACCGTTGTAGCAATACTTCTTTGATTAGTAATAACATCAC**GTGGCTTCGGCTCTT**	55
v8-ZIKV NASBA_Target A 15-40	**GGTGAATTGGGCGTT**TCAACCGATTGAGGGAGGGAAGGTAAATATTGACGGAAAT	55
ZIKV_NASBA_Target B	GATGATCGTTAATGATACAGGACATGAAAC	30
v4-ZIKV NASBA_Target B 40-15	ACCGTTGTAGCAATACTTCTTTGATTAGTAATAACATCAC**GTTTCATGTCCTGTA**	55
v8-ZIKV NASBA_Target B 15-40	**TCATTAACGATCATC**TCAACCGATTGAGGGAGGGAAGGTAAATATTGACGGAAAT	55

Table S9. DNA template, primers, targets and the corresponding detector ssDNA for SARS-CoV-2 RNA detection.

Name	Sequence (5′-3′)	Len.
DNA template	TGGGGTTTTACAGGTAACCTACAAAGCAACCATGATCTGTATTGTCAAGTCCATGGTA ATGCACATGTAGCTAGTTGTGATGCAATCATGACTAGGTGTCTAGCTGTCCACGAGTG CTTTGTTAAGCGTGTT	132
SARS-CoV-2 RNA fragment	UGGGGUUUUACAGGUAACCUACAAAGCAACCAUGAUCUGUAUUGUCAAGUCCAUGGUA AUGCACAUGUAGCUAGUUGUGAUGCAAUCAUGACUAGGUGUCUAGCUGUCCACGAGUG CUUUGUUAAGCGUGUU	132
Forward primer	**AATTCTAATACGACTCACTATAGGGAGAAGG**TGGGGTTTTACRGGTAACCT	55
Reverse primer	AACACGCTTAACAAAGCACTC	30
Target1	CCATGATCTGTATTGTCAAGTCCATGGTAA	
T1-v4-COVID19 40-15	ACCGTTGTAGCAATACTTCTTTGATTAGTAATAACATCAC**TTACCATGGACTTGA**	55
T1-V8-COVID19 15-40	**CAATACAGATCATGG**TCAACCGATTGAGGGAGGGAAGGTAAATATTGACGGAAAT	55
Target2	ATGCAATCATGACTAGGTGTCTAGCTGTCC	
T2-v4-COVID19 40-15	ACCGTTGTAGCAATACTTCTTTGATTAGTAATAACATCACGGACAGCTAGACACC	55
T2-V8-COVID19 15-40	TAGTCATGATTGCATTCAACCGATTGAGGGAGGGAAGGTAAATATTGACGGAAAT	55

Table S10. Backbone and basic variable oligos for the construction of nanoswitches and other oligos.

**Backbone oliogs**		
#	**Sequence (5′-3′)**	**Length**
1	AGAGCATAAAGCTAAATCGGTTGTACCAAAAACATTATGACCCTGTAATACTTTTGCGGG	60
2	AGAAGCCTTTATTTCAACGCAAGGATAAAAATTTTTAGAACCCTCATATATTTTAAATGC	60
3	AATGCCTGAGTAATGTGTAGGTAAAGATTCAAAAGGGTGAGAAAGGCCGGAGACAGTCAA	60
4	ATCACCATCAATATGATATTCAACCGTTCTAGCTGATAAATTAATGCCGGAGAGGGTAGC	60
5	TATTTTTGAGAGATCTACAAAGGCTATCAGGTCATTGCCTGAGAGTCTGGAGCAAACAAG	60
6	AGAATCGATGAACGGTAATCGTAAAACTAGCATGTCAATCATATGTACCCCGGTTGATAA	60
7	TCAGAAAAGCCCCAAAAACAGGAAGATTGTATAAGCAAATATTTAAATTGTAAACGTTAA	60
8	TATTTTGTTAAAATTCGCATTAAATTTTTGTTAAATCAGCTCATTTTTTAACCAATAGGA	60
9	ACGCCATCAAAAATAATTCGCGTCTGGCCTTCCTGTAGCCAGCTTTCATCAACATTAAAT	60
10	GGATAGGTCACGTTGGTGTAGATGGGCGCATCGTAACCGTGCATCTGCCAGTTTGAGGGG	60
11	ACGACGACAGTATCGGCCTCAGGAAGATCGCACTCCAGCCAGCTTTCCGGCACCGCTTCT	60
12	GGTGCCGGAAACCAGGCAAAGCGCCATTCGCCATTCAGGCTGCGCAACTGTTGGGAAGGG	60
13	CGATCGGTGCGGGCCTCTTCGCTATTACGCCAGCTGGCGAAAGGGGGATGTGCTGCAAGG	60
14	CGATTAAGTTGGGTAACGCCAGGGTTTTCCCAGTCACGACGTTGTAAAACGACGGCCAGT	60
15	GCCAAGCTTGCATGCCTGCAGGTCGACTCTAGAGGATCCCCGGGTACCGAGCTCGAATTC	60
16	GTAATCATGGTCATAGCTGTTTCCTGTGTGAAATTGTTATCCGCTCACAATTCCACACAA	60
17	CATACGAGCCGGAAGCATAAAGTGTAAAGCCTGGGGTGCCTAATGAGTGAGCTAACTCAC	60
18	ATTAATTGCGTTGCGCTCACTGCCCGCTTTCCAGTCGGGAAACCTGTCGTGCCAGCTGCA	60
19	TTAATGAATCGGCCAACGCGCGGGGAGAGGCGGTTTGCGTATTGGGCGCCAGGGTGGTTT	60
20	GTTGCAGCAAGCGGTCCACGCTGGTTTGCCCCAGCAGGCGAAAATCCTGTTTGATGGTGG	60
21	TTCCGAAATCGGCAAAATCCCTTATAAATCAAAAGAATAGCCCGAGATAGGGTTGAGTGT	60

22	TGTTCCAGTTTGGAACAAGAGTCCACTATTAAAGAACGTGGACTCCAACGTCAAAGGGCG	60
23	AAAAACCGTCTATCAGGGCGATGGCCCACTACGTGAACCATCACCCAAATCAAGTTTTTT	60
24	GGGGTCGAGGTGCCGTAAAGCACTAAATCGGAACCCTAAAGGGAGCCCCCGATTTAGAGC	60
25	TTGACGGGGAAAGCCGGCGAACGTGGCGAGAAAGGAAGGGAAGAAAGCGAAAGGAGCGGG	60
26	CGCTAGGGCGCTGGCAAGTGTAGCGGTCACGCTGCGCGTAACCACCACACCCGCCGCGCT	60
27	TAATGCGCCGCTACAGGGCGCGTACTATGGTTGCTTTGACGAGCACGTATAACGTGCTTT	60
28	CCTCGTTAGAATCAGAGCGGGAGCTAAACAGGAGGCCGATTAAAGGGATTTTAGACAGGA	60
29	ACGGTACGCCAGAATCCTGAGAAGTGTTTTTATAATCAGTGAGGCCACCGAGTAAAAGAG	60
30	TTGCCTGAGTAGAAGAACTCAAACTATCGGCCTTGCTGGTAATATCCAGAACAATATTAC	60
31	CGCCAGCCATTGCAACAGGAAAAACGCTCATGGAAATACCTACATTTTGACGCTCAATCG	60
32	TCTGAAATGGATTATTTACATTGGCAGATTCACCAGTCACACGACCAGTAATAAAAGGGA	60
33	CATTCTGGCCAACAGAGATAGAACCCTTCTGACCTGAAAGCGTAAGAATACGTGGCACAG	60
34	ACAATATTTTTGAATGGCTATTAGTCTTTAATGCGCGAACTGATAGCCCTAAAACATCGC	60
35	CATTAAAAATACCGAACGAACCACCAGCAGAAGATAAAACAGAGGTGAGGCGGTCAGTAT	60
36	TAACACCGCCTGCAACAGTGCCACGCTGAGAGCCAGCAGCAAATGAAAAATCTAAAGCAT	60
37	CACCTTGCTGAACCTCAAATATCAAACCCTCAATCAATATCTGGTCAGTTGGCAAATCAA	60
38	CAGTTGAAAGGAATTGAGGAAGGTTATCTAAAATATCTTTAGGAGCACTAACAACTAATA	60
39	GATTAGAGCCGTCAATAGATAATACATTTGAGGATTTAGAAGTATTAGACTTTACAAACA	60
40	CATTATCATTTTGCGGAACAAAGAAACCACCAGAAGGAGCGGAATTATCATCATATTCCT	60
41	GATTATCAGATGATGGCAATTCATCAATATAATCCTGATTGTTTGGATTATACTTCTGAA	60
42	TAATGGAAGGGTTAGAACCTACCATATCAAAATTATTTGCACGTAAAACAGAAATAAAGA	60
43	AATTGCGTAGATTTTCAGGTTTAACGTCAGATGAATATACAGTAACAGTACCTTTTACAT	60
44	CGGGAGAAACAATAACGGATTCGCCTGATTGCTTTGAATACCAAGTTACAAAATCGCGCA	60
45	GAGGCGAATTATTCATTTCAATTACCTGAGCAAAAGAAGATGATGAAACAAACATCAAGA	60
46	AAACAAAATTAATTACATTTAACAATTTCATTTGAATTACCTTTTTTAATGGAAACAGTA	60
47	CATAAATCAATATATGTGAGTGAATAACCTTGCTTCTGTAAATCGTCGCTATTAATTAAT	60
48	TTTCCCTTAGAATCCTTGAAAACATAGCGATAGCTTAGATTAAGACGCTGAGAAGAGTCA	60
49	ATAGTGAATTTATCAAAATCATAGGTCTGAGAGACTACCTTTTTAACCTCCGGCTTAGGT	60
50	GAAAACTTTTTCAAATATATTTTAGTTAATTTCATCTTCTGACCTAAATTTAATGGTTTG	60
51	AAATACCGACCGTGTGATAAATAAGGCGTTAAATAAGAATAAACACCGGAATCATAATTA	60
52	CTAGAAAAAGCCTGTTTAGTATCATATGCGTTATACAAATTCTTACCAGTATAAAGCCAA	60
53	CGCTCAACAGTAGGGCTTAATTGAGAATCGCCATATTTAACAACGCCAACATGTAATTTA	60
54	GGCAGAGGCATTTTCGAGCCAGTAATAAGAGAATATAAAGTACCGACAAAAGGTAAAGTA	60
55	ATTCTGTCCAGACGACGACAATAAACAACATGTTCAGCTAATGCAGAACGCGCCTGTTTA	60
56	TCAACAATAGATAAGTCCTGAACAAGAAAAATAATATCCCATCCTAATTTACGAGCATGT	60
57	AGAAACCAATCAATAATCGGCTGTCTTTCCTTATCATTCCAAGAACGGGTATTAAACCAA	60
58	GTACCGCACTCATCGAGAACAAGCAAGCCGTTTTTATTTTCATCGTAGGAATCATTACCG	60
59	CGCCCAATAGCAAGCAAATCAGATATAGAAGGCTTATCCGGTATTCTAAGAACGCGAGGC	60
60	ATTTTGCACCCAGCTACAATTTTATCCTGAATCTTACCAACGCTAACGAGCGTCTTTCCA	60
61	GAGCCTAATTTGCCAGTTACAAAATAAACAGCCATATTATTTATCCCAATCCAAATAAGA	60
62	AACGATTTTTTGTTTAACGTCAAAAATGAAAATAGCAGCCTTTACAGAGAGAATAACATA	60
63	AAAACAGGGAAGCGCATTAGACGGGAGAATTAACTGAACACCCTGAACAAAGTCAGAGGG	60
64	TAATTGAGCGCTAATATCAGAGAGATAACCCACAAGAATTGAGTTAAGCCCAATAATAAG	60

65	AGCAAGAAACAATGAAATAGCAATAGCTATCTTACCGAAGCCCTTTTTAAGAAAAGTAAG	60
66	CAGATAGCCGAACAAAGTTACCAGAAGGAAACCGAGGAAACGCAATAATAACGGAATACC	60
67	CAAAAGAACTGGCATGATTAAGACTCCTTATTACGCAGTATGTTAGCAAACGTAGAAAAT	60
68	ACATACATAAAGGTGGCAACATATAAAAGAAACGCAAAGACACCACGGAATAAGTTTATT	60
69	TTGTCACAATCAATAGAAAATTCATATGGTTTACCAGCGCCAAAGACAAAAGGGCGACAT	60
70	TCACCGTCACCGACTTGAGCCATTTGGGAATTAGAGCCAGCAAAATCACCAGTAGCACCA	60
71	TTACCATTAGCAAGGCCGGAAACGTCACCAATGAAACCATCGATAGCAGCACCGTAATCA	60
72	GTAGCGACAGAATCAAGTTTGCCTTTAGCGTCAGACTGTAGCGCGTTTTCATCGGCATTT	60
73	TCGGTCATAGCCCCCTTATTAGCGTTTGCCATCTTTTCATAATCAAAATCACCGGAACCA	60
74	GAGCCACCACCGGAACCGCCTCCCTCAGAGCCGCCACCCTCAGAACCGCCACCCTCAGAG	60
75	CCACCACCCTCAGAGCCGCCACCAGAACCACCACCAGAGCCGCCGCCAGCATTGACAGGA	60
76	GGTTGAGGCAGGTCAGACGATTGGCCTTGATATTCACAAACAAATAAATCCTCATTAAAG	60
77	CCAGAATGGAAAGCGCAGTCTCTGAATTTACCGTTCCAGTAAGCGTCATACATGGCTTTT	60
78	GATGATACAGGAGTGTACTGGTAATAAGTTTTAACGGGGTCAGTGCCTTGAGTAACAGTG	60
79	CCCGTATAAACAGTTAATGCCCCCTGCCTATTTCGGAACCTATTATTCTGAAACATGAAA	60
80	CCAGGCGGATAAGTGCCGTCGAGAGGGTTGATATAAGTATAGCCCGGAATAGGTGTATCA	60
81	CCGTACTCAGGAGGTTTAGTACCGCCACCCTCAGAACCGCCACCCTCAGAACCGCCACCC	60
82	TCAGAGCCACCACCCTCATTTTCAGGGATAGCAAGCCCAATAGGAACCCATGTACCGTAA	60
83	CACTGAGTTTCGTCACCAGTACAAACTACAACGCCTGTAGCATTCCACAGACAGCCCTCA	60
84	TAGTTAGCGTAACGATCTAAAGTTTTGTCGTCTTTCCAGACGTTAGTAAATGAATTTTCT	60
85	GTATGGGATTTTGCTAAACAACTTTCAACAGTTTCAGCGGAGTGAGAATAGAAAGGAACA	60
86	ACTAAAGGAATTGCGAATAATAATTTTTTCACGTTGAAAATCTCCAAAAAAAAGGCTCCA	60
87	AAAGGAGCCTTTAATTGTATCGGTTTATCAGCTTGCTTTCGAGGTGAATTTCTTAAACAG	60
88	CTTGATACCGATAGTTGCGCCGACAATGACAACAACCATCGCCCACGCATAACCGATATA	60
89	TTCGGTCGCTGAGGCTTGCAGGGAGTTAAAGGCCGCTTTTGCGGGATCGTCACCCTCAGC	60
90	CTTTTTCATGAGGAAGTTTCCATTAAACGGGTAAAATACGTAATGCCACTACGAAGGCAC	60
91	CAACCTAAAACGAAAGAGGCAAAAGAATACACTAAAACACTCATCTTTGACCCCCAGCGA	60
92	TTATACCAAGCGCGAAACAAAGTACAACGGAGATTTGTATCATCGCCTGATAAATTGTGT	60
93	CGAAATCCGCGACCTGCTCCATGTTACTTAGCCGGAACGAGGCGCAGACGGTCAATCATA	60
94	AGGGAACCGAACTGACCAACTTTGAAAGAGGACAGATGAACGGTGTACAGACCAGGCGCA	60
95	TAGGCTGGCTGACCTTCATCAAGAGTAATCTTGACAAGAACCGGATATTCATTACCCAAA	60
96	TCAACGTAACAAAGCTGCTCATTCAGTGAATAAGGCTTGCCCTGACGAGAAACACCAGAA	60
97	CGAGTAGTAAATTGGGCTTGAGATGGTTTAATTTCAACTTTAATCATTGTGAATTACCTT	60
98	ATGCGATTTTAAGAACTGGCTCATTATACCAGTCAGGACGTTGGGAAGAAAAATCTACGT	60
99	TAATAAAACGAACTAACGGAACAACATTATTACAGGTAGAAAGATTCATCAGTTGAGATT	60
100	TAAGAGCAACACTATCATAACCCTCGTTTACCAGACGACGATAAAAACCAAAATAGCGAG	60
101	AGGCTTTTGCAAAAGAAGTTTTGCCAGAGGGGGTAATAGTAAAATGTTTAGACTGGATAG	60
102	CGTCCAATACTGCGGAATCGTCATAAATATTCATTGAATCCCCCTCAAATGCTTTAAACA	60
103	GTTCAGAAAACGAGAATGACCATAAATCAAAAATCAGGTCTTTACCCTGACTATTATAGT	60
104	CAGAAGCAAAGCGGATTGCATCAAAAAGATTAAGAGGAAGCCCGAAAGACTTCAAATATC	60
105	GCGTTTTAATTCGAGCTTCAAAGCGAACCAGACCGGAAGCAAACTCCAACAGGTCAGGAT	60
106	TAGAGAGTACCTTTAATTGCTCCTTTTGATAAGAGGTCATTTTTGCGGATGGCTTAGAGC	60
107	TTAATTGCTGAATATAATGCTGTAGCTCAACATGTTTTAAATATGCAACTAAAGTACGGT	60

108	GTCTGGAAGTTTCATTCCATATAACAGTTGATTCCCAATTCTGCGAACGAGTAGATTTAG	60
109	TTTGACCATTAGATACATTTCGCAAATGGTCAATAACCTGTTTAGCTAT	49

**Variable oligos**		
**Name**	**Sequence (5′-3′)**	**Length**
Var 1	AACATCCAATAAATCATACAGGCAAGGCAAAGAATTAGCAAAATTAAGCAATAAAGCCTC	60
Var 2	GTGAGCGAGTAACAACCCGTCGGATTCTCCGTGGGAACAAACGGCGGATTGACCGTAATG	60
Var 3	TTCTTTTCACCAGTGAGACGGGCAACAGCTGATTGCCCTTCACCGCCTGGCCCTGAGAGA	60
**Var 4**	TCTGTCCATCACGCAAATTA**ACCGTTGTAGCAATACTTCTTTGATTAGTAATAACATCAC**	**60**
Var 5	**ATTCGACAACTCGTATTAAATCCTTTGCCCGAACGTTATT**AATTTTAAAAGTTTGAGTAA	60
**Var 6**	**TGGGTTATATAACTATATGTAAATGCTGATGCAAATCCAA**TCGCAAGACAAAGAACGCGA	60
**Var 7**	**GTTTTAGCGAACCTCCCGACTTGCGGGAGGTTTTGAAGCC**TTAAATCAAGATTAGTTGCT	60
**Var 8**	**TCAACCGATTGAGGGAGGGAAGGTAAATATTGACGGAAAT**TATTCATTAAAGGTGAATTA	**60**
Var 9	GTATTAAGAGGCTGAGACTCCTCAAGAGAAGGATTAGGATTAGCGGGGTTTTGCTCAGTA	60
Var 10	AGCGAAAGACAGCATCGGAACGAGGGTAGCAACGGCTACAGAGGCTTTGAGGACTAAAGA	60
Var 11	TAGGAATACCACATTCAACTAATGCAGATACATAACGCCAAAAGGAATTACGAGGCATAG	60
Var 12	ATTTTCATTTGGGGCGCGAGCTGAAAAGGTGGCATCAATTCTACTAATAGTAGTAGCATT	60

**Filler oligos**		
**Name**	**Sequence (5′-3′)**	**Length**
Var 4 filler	TCTGTCCATCACGCAAATTA	20
Var 5 filler	AATTTTAAAAGTTTGAGTAA	20
Var 6 filler	TCGCAAGACAAAGAACGCGA	20
Var 7 filler	TCGCAAGACAAAGAACGCGA	20
Var 8 filler	TATTCATTAAAGGTGAATTA	20
Var 9 filler	TAGCGGGGTTTTGCTCAGTA	20

**Other oligos**		
Blocking	TCTCATGGCCCTTC	14
BtsCI cut site oligo	CTACTAATAGTAGTAGCATTAACATCCAATAAATCATACA	40
